# Genome-wide DNA methylation map of human neutrophils reveals widespread inter-individual epigenetic variation

**DOI:** 10.1038/srep17328

**Published:** 2015-11-27

**Authors:** Aniruddha Chatterjee, Peter A. Stockwell, Euan J. Rodger, Elizabeth J. Duncan, Matthew F. Parry, Robert J. Weeks, Ian M. Morison

**Affiliations:** 1 Department of Pathology, Dunedin School of Medicine, University of Otago, 270 Great King Street, Dunedin 9054, New Zealand; 2 Gravida: National Centre for Growth and Development, 2-6 Park Ave, Grafton, Auckland 1142, New Zealand; 3 Department of Biochemistry, University of Otago, 710 Cumberland Street, Dunedin 9054, New Zealand; 4 Laboratory for Evolution and Development, Department of Biochemistry, University of Otago, 710 Cumberland Street, Dunedin 9054, New Zealand; 5 Department of Mathematics and Statistics, University of Otago, P.O. Box 56, Dunedin, 9054, New Zealand

## Abstract

The extent of variation in DNA methylation patterns in healthy individuals is not yet well documented. Identification of inter-individual epigenetic variation is important for understanding phenotypic variation and disease susceptibility. Using neutrophils from a cohort of healthy individuals, we generated base-resolution DNA methylation maps to document inter-individual epigenetic variation. We identified 12851 autosomal inter-individual variably methylated fragments (iVMFs). Gene promoters were the least variable, whereas gene body and upstream regions showed higher variation in DNA methylation. The iVMFs were relatively enriched in repetitive elements compared to non-iVMFs, and were associated with genome regulation and chromatin function elements. Further, variably methylated genes were disproportionately associated with regulation of transcription, responsive function and signal transduction pathways. Transcriptome analysis indicates that iVMF methylation at differentially expressed exons has a positive correlation and local effect on the inclusion of that exon in the mRNA transcript.

Methylation of DNA is a mechanism for regulating gene function in all vertebrates. It has a role in gene silencing, tissue differentiation, genomic imprinting, chromosome X inactivation, phenotypic plasticity, and disease susceptibility^1^,^2^. Aberrant DNA methylation has been implicated in the pathogenesis of several human diseases, especially cancer^3^,^4^,^5^.

Variation in DNA methylation patterns in healthy individuals has been hypothesised to alter human phenotypes including susceptibility to common diseases^6^ and response to drug treatments^7^. The impact of epigenetic variation in modulating gene expression and phenotypic traits has been demonstrated in cloned animals^8^ and model organisms^9^.

Although the potential effects of DNA methylation variation has been speculated^10^, firm evidence of variable methylation between healthy human individuals is relatively limited. Recently, variable methylation has been described in different ethnic population^11^,^12^,^13^,^14^. Previous documentation of inter-individual variation in DNA methylation has been affected by the use of mixed cell types in cord or whole blood 12,15,16 or peripheral blood leukocytes^17^. Different cell types exhibit distinct DNA methylation patterns^18^,^19^ and these differences contribute substantially to inter-individual DNA methylation^20^. A recent large-scale epigenomic map revealed substantial variation between human tissue types, further suggesting use of mixed cell types can confound discovery of inter-individual variation^21^. Few studies have attempted to investigate DNA methylation variation in an individual cell type^22^,^23^.

Here we present single-nucleotide resolution DNA methylation maps from 11 healthy individuals, using Reduced Representation Bisulfite Sequencing (RRBS). We choose neutrophils as these are an accessible, abundant and homogeneous cell type. Implementing a novel fragment-based analysis approach^24^, we identified genomic regions that showed significant inter-individual variation in DNA methylation. We explored methylation variation in different elements of the genome (promoters, gene body and regions far upstream of the gene) and integrated them with gene regulatory features (such as transcription factor binding sites (TFBS), histone marks and enhancers) and repetitive elements to gain a perspective on the potential role of methylation variation in genome regulation. Further, we determined that variable methylation is associated with differential gene expression and exon usage, providing a mechanism by which variable methylation might affect the phenotype of these individuals.

## Results

### Features of neutrophil methylome

We used enriched neutrophils (median purity = 96%) from the peripheral blood of 11 healthy individuals to generate DNA methylation maps (Supplementary Table S1). A total of 12 neutrophil methylomes, including a technical replicate, were generated using RRBS and 340 million sequenced reads were obtained. Unique alignment efficiency ranged from 55.5% to 72.4% (median = 67%, Supplementary Table S2). The distribution of read coverage of CpG sites suggested that PCR-induced amplification bias of the libraries was negligible (Supplementary Fig. S1). The median bisulfite conversion rate was calculated to be >98%, assuming that all non-CpG methylation was due to inefficient bisulfite conversion. As expected the neutrophils showed a bimodal distribution of DNA methylation (Supplementary Fig. S2). The level of DNA methylation was significantly lower in promoters (median = 3.0%) and core CpG islands (CGI) (median = 2.4%) than in gene bodies (median = 17.4%) and regions upstream from genes (median = 87.8%) and CGI shore (median = 88.2%) and shelf (median = 82.3%) (ANOVA test, *P*-value <2 × 10^−16^; Supplementary Fig. S3-S4). Exons showed significantly lower levels of methylation compared to introns (9.9% vs. 83.3% median methylation; Supplementary Fig. S5). To assess the technical reproducibility, we compared methylation of fragments of two replicates by using a Bland-Altman plot, in which the 95% limits of agreement were −11.7% to 11.5% methylation. The graphical presentation of agreement (Supplementary Fig. S6) demonstrates high reproducibility of RRBS data, in agreement with previous reports^25^,^26^.

### Identification of inter-individual variably methylated fragments (iVMFs)

To investigate inter-individual DNA methylation variation we used MspI fragments (40–220 bp in size, median size of the fragments = 104 bp, average fragment size = 111.5) as the unit of analysis^24^. To filter fragments that were suitable for analysis, first we selected the fragments having 10 or more reads at ≥2 CpG sites in each individual (Supplementary Table S3). Of the predicted *in silico* 647,626 MspI fragments within an RRBS genome (40–220 bp sized fragments), the number of qualifying fragments per individual ranged from 115,141 to 347,536. From these qualifying fragments 64,934 MspI fragments (containing 432,957 CpG sites) satisfied the inclusion criteria in at least 9 of the 11 sequenced individuals. Henceforth, these are referred to as the “analysed fragments”. A Chi-square distribution test was then performed on the analysed fragments across these individuals to identify fragments with the largest variability (Supplementary Fig. S7).

We identified 14,489 inter-individual variably methylated fragments (iVMFs) that exceeded our significance threshold (Bonferroni adjusted cut off *P-*value = 1.54 × 10^−8^). These iVMFs comprised 22.3% of the analysed fragments, and 2.2% of the *in silico* MspI-digested reduced representation (RR) genome (40–220 bp fragments). These iVMFs contained 92,013 CpG sites, which comprised 21.3% of those contained within the analysed genome and 2.3% of those contained within the *in silico* MspI digested RR genome.

### Comparison of Chi-square test with other methods for detecting variable methylation

**Figure 1:**
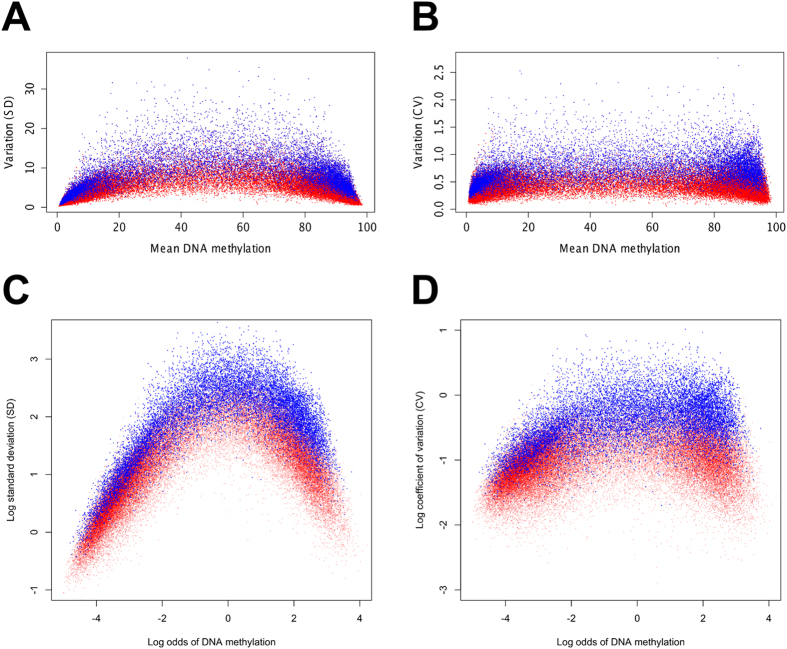
Visualisation of variable methylation: comparison of SD, CV and transformation methods with Chi-squared test. (**A**) Standard deviation (SD) vs. mean DNA methylation. (**B**) Coefficient of variation (CV) vs. mean DNA methylation. CV was calculated as CV = SD divided by the square root of (mean*(100-mean)/n), where n is the number individuals sampled. (**C**) The x-axis shows log odds of methylation, defined as log mean/(100-mean). The y-axis shows log of SD. (**D**) Log odds of methylation vs. log of CV. Red dots represent all autosomal fragments analysed including the iVMFs. Autosomal iVMFs (blue dots) are overlaid on all analysed fragments.

Although the distribution of methylation data is not well characterised, other groups have measured methylation variability using standard deviation (SD) methods^13^,^16^. We compared the Chi-square statistics with SD to assess how these two methods detect variability in our cohort. For this comparison we first plotted the mean DNA methylation against the SD for all the analysed fragments (plotted in red in Fig. 1A), then overlaid the iVMFs (plotted in blue) as detected by the Chi-square test. The expectation was that the iVMFs would show higher SD compared to the total analysed fragments. In fact, the median SD of the iVMFs was 7.68 compared to 3.55 for all fragments analysed (the median SD of the non-variable fragments was 2.99). However, the iVMFs with very high or very low levels of methylation had low SDs. We performed a similar comparison using a coefficient of variation (CV). We calculated a modified CV to account for the DNA methylation scale of 0 to 100% (Fig. 1B). Although the use of CV slightly improved the separation of iVMFs from analysed fragments compared to SD, the iVMFs with high and low methylation still showed relatively low CV, compared to iVMFs with intermediate methylation.

DNA methylation close to 0 or 100% necessarily leads to a low SD as methylation tends to be tightly clustered around these extremes. To enhance visualisation we log-transformed the SD or CV values and performed a logit transformation (log odds) of methylation. After the transformation, the iVMFs showed much more obvious separation from the non-variable fragments based on (log) SD (Fig. 1C) or (log) modified CV (Fig. 1D).

### Chromosome-wise distribution of iVMFs

**Figure 2:**
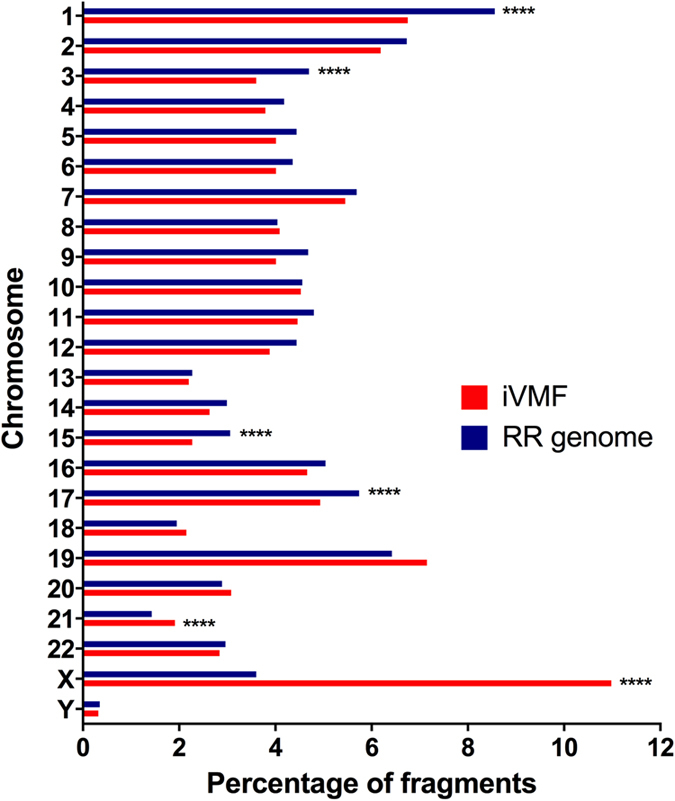
Chromosome-wide fragment distribution of iVMFs and the *in silico* RR genome. For RR genome, the % of RR MspI fragments was calculated for each chromosome (number of fragment in RR genome of 40–220 bp = 647,626). Statistical significance was calculated with Chi square test (with Yates correction, ****P < 0.0001).

Chromosome-wise distribution of iVMFs and *in silico* RR genome fragments revealed that neither the iVMFs nor the RR genome follow the length distribution of the chromosomes, possibly due to the different frequencies of CCGG sites within them. For most of the chromosomes (17 out of 24), the proportion of iVMFs per chromosome was less than that for the RR genome, and of these chromosomes 1, 3, 15 and 17 showed significantly lower levels of variable methylation (*P* < 0.0001, Chi-square test). However, chromosomes 21 and X showed significantly higher levels of variation (*P* < 0.0001) (Fig. 2). Chromosome X showed a striking increase in iVMFs (11%) compared to the distribution of the RR genome (3.6%). As our cohort included six females this result was expected and served as a useful internal control since in females one X chromosome is inactivated by heavy methylation. 55.2% of the iVMFs in the X chromosome were in gene bodies, concordant with the finding that the active X chromosome exhibits significant difference in gene body methylation between male and female^27^. We attempted to further address whether inter-individual variation identified could be is a by-product of sex differences and performed differential methylation analysis between male and female groups (with ANOVA, using DMAP^24^ package). After multiple test correction (using Bonferroni correction at significance level of 0.05), none of the analysed 41464 fragments remained significant between male and female groups. Next, ignoring *P*-value, we analysed fragments that showed a mean methylation difference of 20% or higher between male and female groups and identified 110 fragments. We found 102 fragments were hypermethylated in females compared to males (median difference = 22%) and only 8 fragments were hypermethylated in male subjects compared to females (median difference = 25%) and 91 of these 110 fragments (83%) were located in chromosome X. These data suggest that number of differentially methylated fragments between our male and female groups is negligible and autosomal chromosomes are least likely to be affected by sex specific variation in healthy individuals. The allosomal iVMFs were excluded to avoid potential sex-specific effects, resulting in 12,851 autosomal iVMFs for further analysis (Supplementary Table S10).

### Genomic distribution of iVMFs

**Figure 3:**
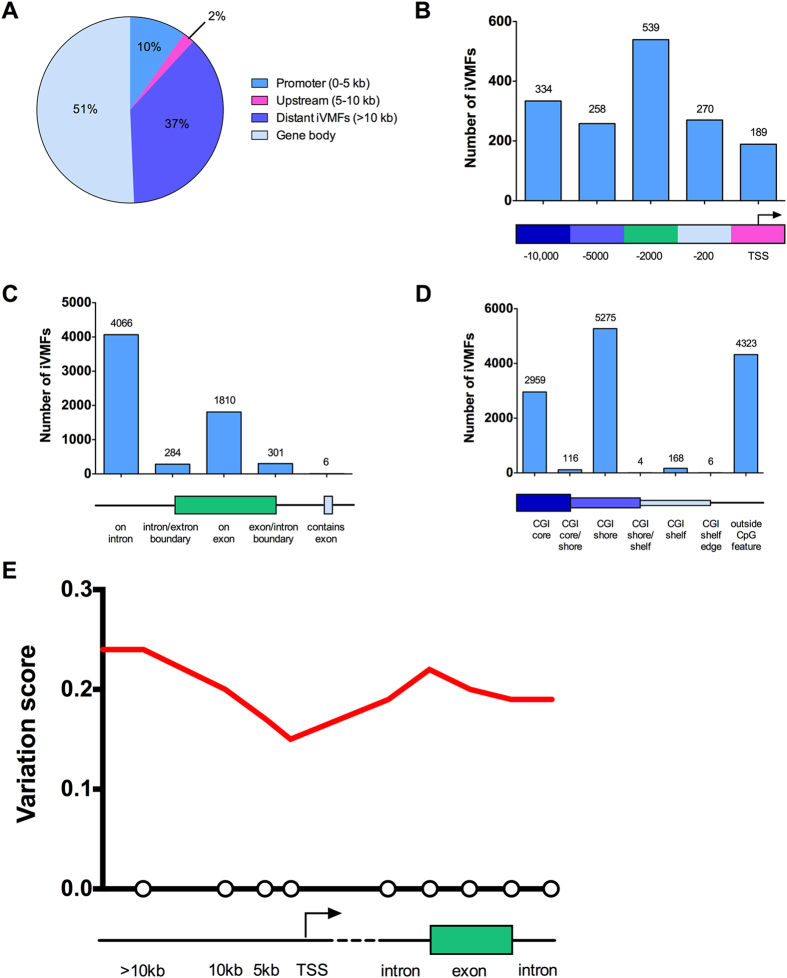
Landscape of iVMFs in different genomic elements. (**A**) Overall distribution of the iVMFs in genomic elements. (**B**) Distribution of the iVMFs around transcription start site and regions upstream from the start of the gene. (**C**) Distribution of the iVMFs in the gene body elements. (**D**) Distribution of the iVMFs in different CpG feature. (**E**) Schematic landscape of DNA methylation variability across the genome. For each feature variability score was calculated as the number of iVMFs/number of analysed fragments for that feature. Each circle on x-axis represents the variation score of a genomic feature.

The autosomal iVMFs were mapped to their nearest protein coding gene (Fig. 3A). Overall, 9.8% of the iVMFs were located in promoters (defined as up to 5 kb upstream from the start of the gene). Within the promoter-associated iVMFs, 15% showed direct overlap with the transcription start site (TSS) of known protein-coding genes (Fig. 3B). Interestingly, 50.4% of the iVMFs reside within the gene body (includes exon, introns and exon/intron or intron/exon boundaries). Of the 6481 gene-body-associated iVMFs, 62.9% were found to be in introns and 28.0% in exons. A small proportion of the iVMFs were found to be in exon/intron and intron/exon boundaries (Fig. 3C). We found 37.2% of the iVMFs were located more than 10 kb upstream from the nearest gene start site (defined as distant iVMFs).

We sought to determine whether this iVMF distribution in genomic features merely reflects the distribution of analysed fragments or whether different elements in the genome biologically differ in methylation variability. Therefore, we assigned a variability score (VS) to each of these genomic features by dividing the number of iVMFs with the number of analysed fragments for that feature. Promoter, gene body and distant regions had a variability score of 0.16, 0.20 and 0.24 respectively; gene promoters were significantly less variable compared to other genomic regions (*P* = 0.0001). Moreover, the variability score showed consistent increments with distance from the promoter (Fig. 3E). The lowest variability score of 0.15 was found in the region of 0 - 2 kb upstream of the transcription start site. Within the gene body, there was no significant difference between the variability within the exons, introns, intron/exon boundaries and exon/intron boundaries.

The distribution of iVMFs within CpG features (CGI core, shore (flanking 2 kb either side of the core CGI) and shelf (flanking 2 kb either side of the CGI shore)) was investigated. 65.3% (8392) of the iVMFs were located within one of these CpG features. It was found that 23.0%, 41.0% and 1.3% of the iVMFs were within CGI cores, CGI shores and CGI shelves respectively (Fig. 3D) similar to the distribution of analysed fragments (24.0%, 39.9% and 1.3% respectively) (Supplementary Table S4).

The mean number of CpG sites per fragment were significantly higher in iVMFs (promoter, gene body and distant fragments) compared to the non-variable fragments (*P* = 0.0001) (Supplementary Table S5), suggesting that although the iVMFs were not enriched for a particular CpG feature, their mean CpG density is higher than the analysed non-variable fragments.

### Genome regulation and variable methylation

To determine whether variable DNA methylation is enriched within regions with particular chromatin states or regulatory roles, we integrated the iVMFs with publicly available data for gene regulatory elements (ENCODE consortium^28^). The promoter, gene body and distant iVMFs were separately analysed in the EpiExplorer platform^29^ to determine their overlap with gene regulatory features. We focused our comparison with the annotated features of the K562 cell line (derived from human chronic myelogenous leukemia)^30^ as, of the nine annotated cell types available, this cell line is the most closely related to neutrophils. However, K562 will show many differences in transcription factor expression, histone marks and regulatory elements than those in neutrophils.”

**Figure 4:**
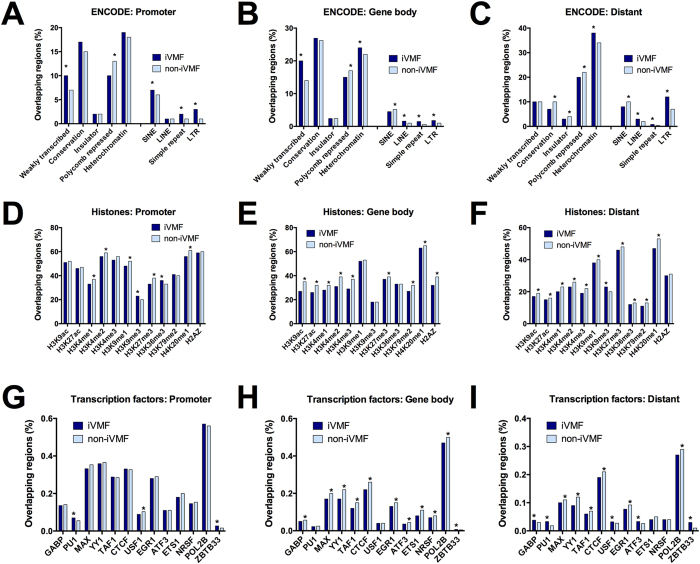
Comparison of the overlap of iVMFs with regulatory feature and chromatin state maps. Overlap of iVMFs was compared with the non-variable fragments for the same feature. (**A–C**) Overlap comparison of all three categories (promote, distant, gene body) of iVMFs with genome regulatory feature. (**D–F**) Overlap comparison with major histone modification marks. (**G–I**) Overlap comparison with major transcription factors. Statistical significance was calculated with Chi-square test (with Yates correction) and features with *P* < 0.05 are indicated by an asterisk.

For this comparison, iVMFs with either a significantly higher or lower proportion (*P* < 0.05) of overlap within a feature compared to non-variable fragments were referred to as either “enriched” or “depleted” respectively (Supplementary Table S11). Promoter and gene body iVMFs were enriched in weakly transcribed regions in the genome. All three categories of iVMFs (distant, promoter and gene body) were depleted in polycomb-repressed regions and were enriched in repetitive elements. Whereas simple repeats and low terminal repeats follow this trend, long interspersed elements (LINE) were only enriched in gene body and distant iVMFs. On the other hand, short interspersed elements (SINE) were enriched in promoter iVMFs but depleted in gene-body and distant iVMFs. Distant iVMFs were depleted in conserved regions and insulator elements (Fig. 4A–C and Supplementary Table S11).

Different histone modification marks have distinct roles and distribution across the genome^31^. We compared the association of iVMFs with 12 major histone modification marks (10 active marks and 2 repressive mark) with that for the non-variable fragments. The promoter iVMFs were depleted for the following active marks: H3K4me1, H3K4me2, H3K9me1 and H4K20me1(as described by Barski *et al.*^32^ and designated as active promoter state by Ernst and Kellis^31^). In addition to these active marks, promoter iVMFs were also depleted for the repressive mark H3K27me3. On the other hand, the promoter iVMFs were enriched for the active mark H3K36me3 and the repressive mark H3K9me3. The gene body iVMFs showed depletion for the majority of the histone marks (for eight active and one repressive mark). The distant iVMFs were also depleted for most histone modifications (9 out of 12), except H3K4me1 (active mark) and H3K9me3 (repressive mark), for which they were significantly enriched (Fig. 4D–F, and Supplementary Table S11).

One of the mechanisms by which DNA methylation can regulate gene expression is by modulating the binding and interaction of transcription factors (TF) with DNA^33^. We analysed the overlap of iVMFs with 20 major transcription factor binding sites. The promoter iVMFs did not show significant association with most transcription factors except in PU1 sites where iVMFs were enriched and in USF1 sites where they were depleted. All three categories of iVMFs (distant, promoter and gene body) were enriched for transcriptional regulator Kaiso. The gene body iVMFs showed depletion for 10 out of 20 TF binding sites and no association with the remaining 9 TFs. The distant iVMFs also showed significant enrichment for several transcription factors binding sites (GABP, PU1, BCLAF1, SRF, SIX5, USF1, ATF3, SIN3AK20, MEF2A) (Fig. 4G–I). It is possible that some of these results could be influenced by genotypic variation between the individuals (see discussion for detail).

### Identification of variably methylated genes

To generate a list of highly variable methylated genes (VMGs), the 12,851 autosomal iVMFs were associated with 6353 protein coding human genes. Then iVMF frequency was calculated, i.e., assigning the number of iVMFs associated with each gene. We found 1298 genes had three or more associated iVMFs (Supplementary Table S6). A variability score was assigned to each of these genes by dividing the number of associated iVMFs by the number of analysed fragments for that gene. Of these 1298 genes, 441 showed a variability score of ≥0.5; i.e., more than half of the analysed fragments were variable (Supplementary Table S12). For this analysis, no limit was imposed on how far upstream an iVMF can lie from these genes.

To identify VMGs present within functionally important regions (promoter, gene body, enhancer and insulator), a variability score was assigned to fragments within each functional category (regions containing <3 iVMFs were not considered). We identified 29, 240, 4 and 8 genes that showed high variability (≥0.5) in their promoter, gene body, enhancer and insulator elements respectively (Table 1 and Supplementary Table S12; 10 genes were included in more than one category, Supplementary Fig. S8).

### Overlap of variable genes with previous genome-wide studies and assessment of tissue specificity in iVMFs

We compared our data with three previously published genome-wide studies in relation to our candidate variable genes. Of the five previously reported human gene-associated metastable epialleles (in peripheral blood leukocytes of rural Gambian children)^17^, one appeared in our list of top variable genes (*ZFYVE28* with 9 iVMFs and variability score = 0.69). The variable nature of *ZFYVE28* was also reported by another independent study^34^. Another metastable epiallele at *PAX8* that was previously validated in human also appeared in our initial list (*PAX8* with 7 iVMFs and variability score = 0.41); however, *PAX8* was not among our top variable genes as its variability score was <0.5.

Feinberg *et al.* reported 212 variably methylated genes in whole blood from their use of comprehensive array-based relative methylation (CHARM) analysis^16^. Of these, 121 were within the promoter region (<5 kb upstream of the gene). Six genes appeared in both lists. This degree of overlap was expected by chance (*P* = 0.60, hypergeometric test). Harris and colleagues previously reported 1013 promoter and gene body associated metastable epiallele genes^34^. When we compared our promoter and gene body associated VMGs (271 genes) with that of Harris, we found highly significant overlap of 43 genes (*P* = 1.75 × 10^−7^, hypergeometric test). Further, two genes were identified that were common between the three independent studies (our study, Feinberg *et al.* and Harris *et al.*; Supplementary Boxes 1–6). The differences in the number of overlapping genes between these studies could be attributed to the mixed cell type used in the previous studies, the platform used for DNA methylation analysis and false discovery rates (FDR).

After performing the Chi-square test, we used a stringent Bonferroni correction to derive a cut-off *P*-value for filtering iVMFs. After applying the Bonferroni correction, a large number of fragments remained significant. Use of different multiple correction method could yield different lists of differentially methylated regions and therefore could explain some of the differences between our study, Feinberg *et al.* and Harris *et al.* studies (see Supplementary Table S9 and description of statistical test to identify inter-individual variably methylated fragments section in Supplementary Information 1 for detailed explanation on statistical methods for methylation analysis).

To assess potential influence of tissue-specific DNA methylation variation in the identified iVMFs, we compared our iVMF data with previously published CpG sites that were differentially methylated among different blood cell types. We have analysed the overlap of our 12851 iVMFs with 1865 differentially methylated CpG sites between different purified blood cell types (CD4+ T cells, CD8+ T cells, NK cells, B cells, monocytes, granulocytes, eosinophils, neutrophils and whole blood) reported from previous genome-wide studies^19^,^35^. We found only 13 out of these 1865 CpG sites (0.7%) overlapped with 11iVMFs. These results further demonstrate that the iVMFs detected in this study are not influenced by tissue-specific methylation variation.

### DNA methylation and gene expression

**Figure 5:**
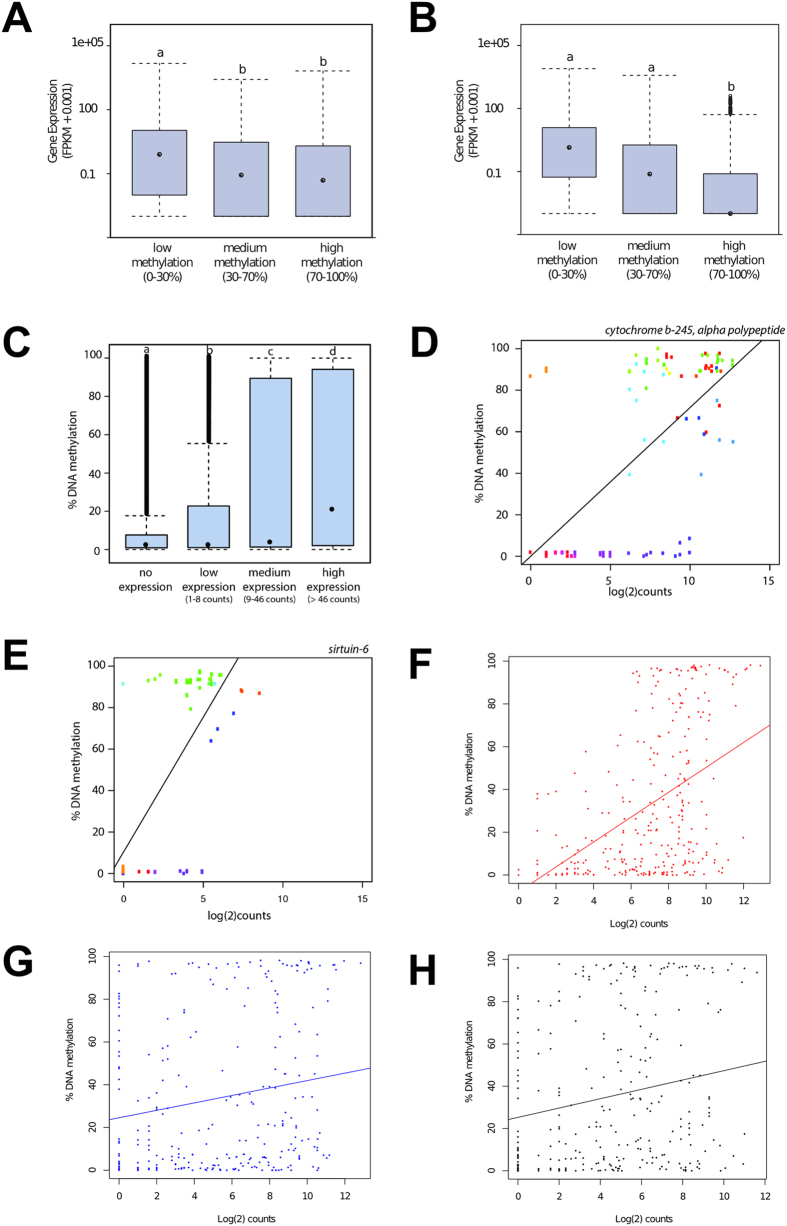
DNA methylation and gene expression relationship. Methylation of CpG island cores (**A**) and promoters (**B**) negatively associated with gene expression (y-axis, plotted as fragment per kb per million reads or FPKM). Bars that do not share a letter are significantly different. For (**B**), DNA methylation of 0 to + 500 bp region (downstream) from transcriptional start site was plotted against expression (see more details in Supplementary Table S7). (**C**) Expression of individual exons is positively correlated with DNA methylation on that exon. For significance tests in the above figures, ANOVA with a Tukey HSD post-hoc test was used and *P* < 0.05 was considered significant). (**D–E**) Two examples of positive correlation between individual exon methylation and gene expression (log 2 of read counts for the exons were plotted in x-axis): cytochrome b-245 alpha polypeptide (*CYBA)* (r = 0.68 and *P* = 8.3 × 10^−9^) and sirtuin6 (*SIRT6*) (r = 0.67 and *P* = 7.5 × 10^−5^) genes. (**F**) Methylation and expression of 78 exons where iVMFs directly overlapped differentially expressed exons, (**G**) exons upstream to these directly overlapped iVMFs and (*H*) exons downstream to these directly overlapped iVMFs.

We performed RNA-Seq on neutrophil RNA from four individuals in our cohort to explore the association of DNA methylation with gene expression. As expected, DNA methylation of core CGIs was negatively associated with expression of corresponding genes: fragments with 0–30% CGI methylation were associated with higher expression compared to the highly methylated (>70%) fragments (Fig. 5A, *P* < 0.05, ANOVA with a Tukey HSD post-hoc test). To investigate the effect of methylation around the TSS on gene expression we used different windows to define promoters. This inverse association between methylation and gene expression was strongest for fragments located 0–500 bp downstream of the start of the gene (*P* = 3.5 × 10^−16^, Fig. 5B). This inverse association became weaker with increased distance upstream of the gene start and >3000 bp upstream of the gene the inverse association was no longer significant (Supplementary Table S7).

At the level of individual exons, highly expressed exons showed higher levels of DNA methylation (Fig. 5C; Supplementary Fig. S9-S10). Two examples were *CYBA* (r = 0.68 and *P* = 8.3 × 10^−9^) and *SIRT6* (r = 0.67 and *P* = 7.5 × 10^−5^) genes (Fig. 5D,E).

Within the four RNA samples, differential expression was identified for 257 genes (false discovery adjusted P ≤ 0.01). Similarly, 1857 genes contained promoter or gene body iVMFs in these four individuals (these iVMFs were re-derived using methylation data for the four individuals as described above for the 11 individuals). When the differentially expressed genes were overlapped with genes that contained iVMFs in their promoter or gene body, 24 genes were in common. Of these 24 genes, three genes contained iVMFs in their promoters (*MAP7D2, PROK2, GATA2*). The other 21 genes contained iVMFs in their body.

Since DNA methylation has been linked with alternative exon usage^36^ we sought to determine the extent to which variation in methylation is associated with exon usage. Using the DEXseq^37^ package we identified exons that were differentially expressed. 78 iVMFs (re-derived for these four individuals) directly overlapped differentially expressed exons. The level of methylation at these iVMFs was positively correlated with inclusion of the exon in the mRNA transcript (r = 0.48, *P* = 2.2 × 10^−16^) (Fig. 5F). As a control, the correlation of iVMF methylation was much weaker with the expression of the upstream (r = 0.17, *P* = 0.004, Fig. 5G) or the downstream exon (r = 0.20, *P* = 0.0008, Fig. 5H). These results suggest that methylation level of an iVMF has a local association with the expression of the iVMF-containing exons but not the adjacent exons.

### Functional enrichment of variably methylated genes

We investigated whether variably methylated genes are disproportionally involved with particular biological functions. For this analysis, we restricted our list to 271 genes that contained iVMFs only in promoter, gene body, enhancer and insulator. The variably methylated genes were significantly enriched for regulation of transcription, gland development, organ morphogenesis and chromatin remodeling related genes. Interestingly, genes with high level of expression in brain genes were significantly enriched in the list of variable genes (Supplementary Table S8 and Supplementary Table S13).

## Discussion

DNA methylation can be interrogated by using different units of analysis (e.g., single CpG sites, fragments, or tiling windows). To investigate DNA methylation variation, we chose MspI fragments as the unit of analysis^24^, since biological relevance tends to be associated with regional methylation and since the use of fragments integrates the information from multiple CpG sites, thereby minimising sampling variation^38^. Use of large tiling windows (e.g. 1000 bp) is a common approach; however for RRBS, the majority of genomic windows will not contain sequenced reads or will have partial inclusion of MspI-digested fragments. Further, if a region is variably methylated, use of a large window might obscure this variation^38^.

Variable promoter DNA methylation has been previously reported in normal individuals^39^. A small proportion of the iVMFs that we identified were in promoters (9.8%) and we showed that the distance from the gene promoter correlated with increased methylation variability, demonstrating that promoters are the least variable regions in the genome. This observation is consistent with the hypothesis that gene promoters are epigenetically conserved, usually having low levels of methylation to prevent mutations and requiring tight regulation for proper functioning of the genome^40^ and therefore cannot accommodate large degree of variation. The variably methylated promoters showed higher CpG density than the non-variable promoters. Promoters with low CpG content generally show high methylation and mainly overlap tissue-specific genes, whereas the high CpG promoters generally have low methylation and are associated with broadly expressed genes^41^,^42^. This suggests that genes associated with promoter iVMFs are more likely to be expressed in many tissues. The variable promoters were enriched in “weakly transcribed regions” consistent with their depletion for highly active histone marks (such as H3K4me1 and H3K4me2).

The extent of genome-scale epigenetic variation and its association with repetitive elements has not been explored. We show significant enrichment of repetitive elements in iVMFs. Simple repeats and LTRs were particularly enriched in all three different categories of iVMFs. SINE elements were enriched in promoter iVMFs but were depleted among gene body and distant iVMFs. Retrotransposons, when close to CpG islands, can act as methylation control centers^43^,^44^ and the overlap of transposable elements with specific enhancer marks can regulate tissue-specific functions^45^. Our analysis shows greater epigenetic variation in repetitive elements compared to other elements of the genome suggesting that repetitive elements perhaps serves as a reservoir to harbor variably methylated loci. Considering more than half of the human genome contains repetitive elements^46^, variation in even a small proportion of these elements has the potential to contribute to epigenetically-induced phenotypic variation. This finding opens up a new avenue for exploring the functional role of epimutations in repetitive elements in healthy individuals.

**Figure 6:**
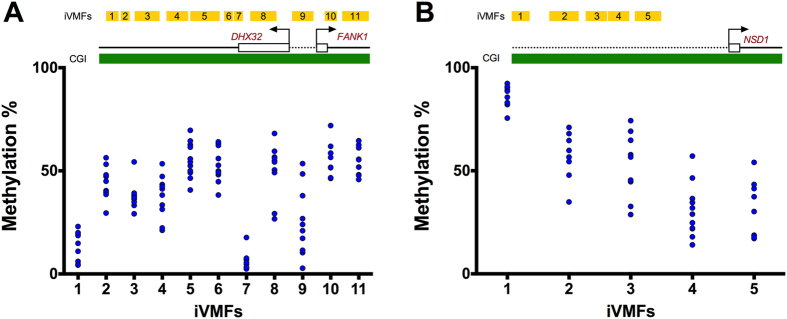
DNA methylation of individuals *for DHX32-FANK1* and *NSD1* gene promoter. (**A**) *DHX32-FANK1* region contained 11 iVMFs within a 942 bp region (chr 10:127585282-127584340) with 3 promoter fragments being in the promoter and 8 fragments being in the first intron of the gene. iVMFs 1–3 reside in the promoter and iVMFs 4–11 in the intron. (**B**) NSD1 promoter contained 5 iVMFs within 543 bp region (chr 5: 176559108-176559651). Each dot represents an individual. iVMFs are drawn in yellow and CpG island (CGI) is drawn in green.

This study and previous reports show that global CpG methylation generally follows a bimodal distribution pattern^47^,^48^. However, at individual CpG sites or in a contig of CpG sites, the distribution of methylation values are not well characterised. We chose to assess variation in DNA methylation using the Chi-square test as it does not assume any particular distribution. Comparative analysis showed that although significantly variable with Chi-square, a substantial proportion of the iVMFs (where average methylation of the individuals were close to 0 or 100%) had low standard deviations. This is exemplified in the top candidate variable region of *DHX32-FANK1* gene (variability score = 1.0) that contained 11 iVMFs (Fig. 6A). For iVMF 5, DNA methylation between individuals ranged from 2.46% to 17.7% (mean = 6.34, SD = 4.09), whereas for iVMF 4 it was 26.7% to 68.1% (mean = 50.5, SD = 12.3). For lowly or highly methylated fragments the methylation variation was detected by Chi-square test but not with SD. We showed that transformation of the SD or CV to log odd ratios of methylation improved the visible separation of variably methylated fragments compared to the non-variable ones. Nevertheless, the Chi-square test treats each methylation count as an independent observation, and correlation between the methylation of neighbouring CpG sites will have an effect on the significance estimation.

**Figure 7:**
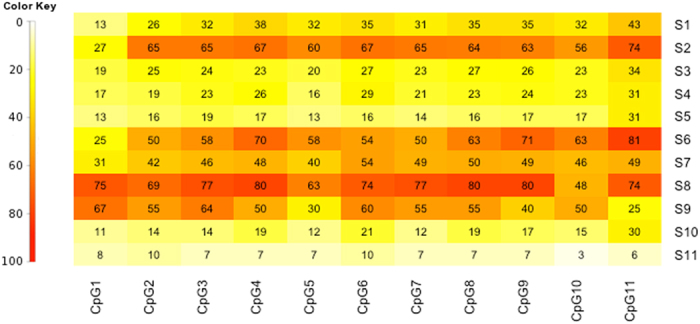
Methylation of adjacent CpG sites in *KRTCAP3*-associated iVMF (chr2: 27665237-27665315). The 79 bp fragment contained 11 CpG sites. CpGs (1–11) are shown in horizontal axis and individuals (S1-S11) are shown in vertical axis. White (0%) to red (100%) represents DNA methylation scale.

In some previous studies it has been demonstrated that adjacent CpG sites are likely to show correlated DNA methylation levels^49^,^50^. However, we noticed for several iVMFs, the methylation pattern between the adjacent CpG sites showed notable variation (Fig. 7 shows an example of such an iVMF). Although we cannot draw conclusions about the biological effect of different methylation at adjacent CpG sites, the methylation status of these CpG sites could, for example, regulate binding of transcription factors^51^ or of the insulator protein CTCF^52^. Further, this observation is from a relatively homogeneous cell population of neutrophils and therefore cannot be attributed to mixed cell types^53^. These data demonstrate the enormous complexity of methylation patterns even between homogenous cells, giving rise to further opportunities for studying cell-to-cell and inter-individual differences.

The functional relationship and the influence of DNA methylation on transcription factor binding have recently begun to be appreciated. A recent analysis reported notable variability in DNA methylation between transcription factor binding sites (TFBS) and gene expression (25,000 TFBS and 164 motifs)^54^. Inter-individual methylation variation could have broad implications in determining TF binding. For example, we observed that promoter and distant iVMFs showed enrichment for PU.1 binding sites. High levels of methylation of PU.1 sites cause silencing of adjacent genes; however, removal of methylation at these sites permits initiation of transcription, as shown in erythroid differentiation and monocyte-to-osteoclast differentiation^55^,^56^. All three categories of iVMFs showed enrichment for transcription regulator Kaiso binding sites. The methylation status of these binding sites has been shown to regulate binding of Kaiso to its target sequence^57^,^58^. Predicting the effects of variable methylation at TFBS is problematic, but epigenetic variability at transcription binding sites is likely to be a mechanism for variable gene transcription between individuals.

Our analysis suggests that the relationship between DNA methylation and gene expression in neutrophils is complex. It has been shown that the DNA methylation status of exons can control binding of insulator elements, such as CTCF, and that this can promote inclusion of a particular exon in a transcript^36^. Here we demonstrate that methylation of exons and introns have different effects on gene expression. A small number of iVMFs overlapped with differentially expressed exons and methylation of these iVMFs were positively correlated with inclusion of the exons in the corresponding transcript. This suggests that variable methylation is associated with exon inclusion, resulting in functional differences in the mRNA transcript and the protein produced. Additional observations will be required to clarify whether methylation promotes inclusion through transcriptional pausing^36^ or alternatively whether it occurs as a consequence of transcribed open chromatin^59^.

Functional enrichment analysis of variably methylated genes indicated that they were enriched for functions relating to regulation of transcription and Chromatin remodelling confirming their potential role in genome regulation. Further, gene ontology analysis suggested that the variable genes are likely to be highly expressed in brain tissue. Epigenetic events have been shown to have roles in memory formation, learning, brain development, establishment of neuronal identity and neurological disorders^60^,^61^,^62^,^63^. In the context of the present study, if epigenetic variation rises at a very early stage of life (before lineage commitment), then these changes will propagate in other cell types. In that case, the majority of early origin epigenetic variation detected in neutrophils could also be observed in brain. In fact, highly correlated DNA methylation patterns between brain and blood has been reported^64^,^65^.

The BLUEPRINT project is in the process of generating epigenomic profiles of at least 100 different blood cell types including neutrophils from healthy and diseased individuals. The analysis modules include whole genome DNA methylation, RNA-Seq, Chip-Seq, Nucleosome detection, DNase-hypersensitive sites and co-occurring transcription factors analysis^66^. Our data provides genome-wide methylation and RNA-Seq profiles from a cohort of normal individuals of different ethnicity. These data are complementary to the BLUEPRINT initiative and provide an additional resource. Further, the BLUEPRINT data provide an opportunity to integrate the multiple levels of epigenomic data to prioritise candidate iVMFs that might have role in non-communicable human disease and explore the functional consequences of variable methylation in larger cohorts. For instance, if epigenetic variation was detected in the promoter of a gene having role in growth, fat metabolism, diabetes or asthma, it will be possible to examine the association of the methylation with the relevant phenotype in a larger cohort to demonstrate the consequences of epipolymorphism. For example, we have identified striking DNA methylation variability in nuclear receptor SET domain containing protein-1 (NSD1) gene promoter (variability score = 1.00, analysed fragment = 5, iVMFs = 5, Fig. 6B) among the individuals. NSD1 has been associated with Sotos syndrome and Weaver syndrome and downregulation of NSD1 protein disrupts the normal activity of genes involved in growth and development^67^,^68^. Evidence for epigenetic inactivation of NSD1 in human cancers also exists^69^. With whole genome data from larger cohort of individuals with good phenotypic description, it will be possible to test whether DNA methylation variation in gene (such as NSD1) are responsible for altering disease susceptibility or phenotypic traits in normal individuals”

In relation to our study it is important to consider the contribution of genetic variation in determining inter-individual DNA methylation variation. Several studies have established that genetic variation (polymorphism) can influence DNA methylation status^26^,^70^,^71^,^72^. It has been shown that DNA methylation patterns of genetically related individuals tend to be similar when compared to unrelated ones^26^ and populations of similar genetic origin were closely related compared to other populations^11^, suggesting genetic influence on epigenetic profiles. However, there is no consensus on the extent to which genetic polymorphisms (e.g., SNPs) influence DNA methylation in an individual. A note here is that studies of association between methylation and SNPs were performed to quantify allele-specific methylation (ASM) variation only. However, recent reports suggest that the number of methylated CpG sites affected by SNPs is small. Hellman *et al.* reported that common SNPs are present in only 10% of the regions that have a difference in DNA methylation between the two alleles in related and unrelated individuals^73^ and independent analysis provided further support for these data^12^. Analysis, including multiple ethnic groups, indicated that genotype can explain ~25% of the variable methylation regions^14^. Importantly, an RRBS study of a three-generation family showed that, among all investigated CpG sites (~1 million with 10 or more sequenced reads), less than 1% of the CpG sites were involved with ASM events^26^ suggesting that influence of SNPs in CpG site methylation in RRBS genome is small. Further, ASM events were found mainly in intergenic regions and outside CpG islands and showed low levels of evolutionary conservation^26^. Further, a recent large-scale analysis indicated although the genotypes of different ethnicity clusters separately and a clear distinction of population can be made by genotype. However, epigenetic profile of the same populations were dispersed and they couldn't be distinguished based on their DNA methylome unlike genotype^14^. This result suggests that potential for DNA methylation variation in individuals is larger than genotypic variation.

In our RRBS experiment the methylation profile of any fragment was expected to be derived from both alleles (ideally 50% from each allele). Therefore, the methylation profile of fragment is a mean of methylation of both alleles. If there is allele-specific methylation bias, then we will not be able to detect it with our method. Genetic vs. epigenetic variation comparison is not possible when methylation information of two alleles are mixed. Additionally, alignment of sequenced reads will ignore C to T SNPs in forward strand reads and G to A SNPs in reverse strand reads, because a C to T match in forward strand reads will be treated as an unmethylated. C to T and G to A SNPs consists 30% of all human SNPs^26^.

MspI cuts the genome at C’CGG sites, so if an individual has a SNP in the recognition site, then the fragment will be missed for that individual. If as a result of a polymorphism, an MspI site has been created, the consequent fragment will not be included in the inter-individual analysis. Nevertheless, evidence of similar DNA methylation patterns in genetically related individuals compared to the unrelated ones suggest a genetic component to epigenetic variation in a normal population^26^. A proportion of iVMFs found in this study could be associated with genetic variation; however distinguishing the genetic and epigenetic variation in identified iVMFs is beyond the scope of the present study and will remain a subject for future research. Further, it will be of broad interest for future research to investigate which regions of the genome genetic variation are more likely to influence variable methylation than others and whether there is sensitive period in development or adulthood that are more vulnerable to accommodate inter-individual DNA methylation variation mediated by genetic variation.

In this study, we selected individuals of diverse genetic origin to maximize the possibility of detecting epigenetic variation. Our results indicate that in a mixed population, epigenetic variation is widespread and is more extensive than previously reported. In conclusion, this work documents epigenetic variation in the human genome and provides a comprehensive resource for future studies aiming to understand the nature and mechanism of phenotypic plasticity due to DNA methylation variation. In addition, these data will be useful to explore complex traits and altered disease susceptibility due to variable DNA methylation pattern in normal individuals.

## Methods

### Ethics statement

Informed consent was obtained from all subjects. Peripheral blood samples were collected from healthy individuals in accordance with the guidelines and approval obtained from the Dunedin Multi-region Ethics Committee, Dunedin, Otago region, New Zealand (approval number: MEC/09/07/068).

### Recruitment of participants

Eleven individuals (5 male and 6 female) were recruited. The individuals represented diverse ethnic backgrounds and ranged in age from 26 to 34 years (see Supplementary Table S1). A technical replicate RRBS library was also included in the analysis.

### Neutrophils isolation and DNA extraction

16 mL peripheral blood was collected from the participating subjects and neutrophils were purified using a Ficoll-Paque method (see details in supplementary methods). DNA was extracted from the neutrophil suspension, using QIAamp DNA mini kit (Qiagen, Hilden, Germany) following the manufacturer’s protocol, with the addition of overnight proteinase K treatment at 55 °C.

### Preparation of RRBS libraries

RRBS provides base-resolution information for about 4 million CpG sites (13.4% of all CpG sites in the genome)^74^ and for more than 23,000 CpG islands (84% of those in the genome)^75^. RRBS libraries were prepared according to our previously published protocols using the TruSeq kit (Illumina)^74^,^76^,^77^,^78^. See supplementary methods for details of library preparation.

### Sequencing RRBS libraries, base-calling and quality assessment

Two RRBS libraries (X9015 and X9006) were sequenced using the Illumina GAII platform and the other libraries were sequenced using the Illumina HiSeq2000 platform. Sequencing was single-ended and the read length was 100 bp. Base-calling was performed with Illumina Real Time Analyzer (RTA) software; however, post-run standardization of the base-calling was performed using Illumina Off-Line Base-calling application where applicable^76^. Quality assessment of sequenced reads and processing was performed as our previously published pipelines (details in supplementary methods).

### Variable methylation analysis

Variable methylation analysis was performed with our in-house Differential Methylation Analysis Package (DMAP)^24^. DMAP contains two main programs (diffmeth and identgenloc). We used MspI fragments as the unit of analysis for identifying variable methylation. Using the diffmeth program, for each individual the MspI fragments that had at least 2 CpG sites, covered by 10 or more sequenced reads were included (F2 t10 switch in the diffmeth program of DMAP tool). Next, a Chi-square test was performed for each fragment, requiring that at least 9 out of 11 individuals contained enough coverage for that fragment. For the Chi square test the proportion of methylation in a fragment is the total number of methylated CpGs in all sequenced reads divided by the total number of CpGs in the reads. The fragments with a *P-*value of ≤ 1.54 × 10^−8^ (Bonferroni corrected, see statistical analysis section) were identified as inter-individual variably methylated fragments (iVMF). For male and female group comparisons, we analysed 41464 fragments (where all 5 male and all 6 female contained at least 10 or more sequenced reads at 2 or more CpGs in a fragment) and performed ANOVA statistical test using DMAP.

### Feature analysis of iVMFs with ENCODE data

Genomic and epigenomic feature analysis of iVMFs was performed using Epiexplorer tool^29^. The genomic co-ordinates of the promoter, gene body and distant iVMFs were separately uploaded to the Epiexplorer server. For comparison, co-ordinates of the analysed non-variable fragments in promoter, gene body and distant regions (within the size range of 40–220 bp) were similarly uploaded and analysed. The fragments that showed strong overlap (i.e., ≥50% of a fragment overlapped with a feature) were filtered for analysis and statistical calculation of enrichment or depletion of iVMFs in a feature was performed. The comparisons described here were based on regulatory features from K562 cells. However, comparison was also made against aggregated data from all nine cell lines and this is referred to as “any tissue” analysis (Supplementary Fig. S11-S13).

### Identification of gene and CpG features for iVMFs

The identgeneloc program of the DMAP tool was used to locate iVMFs to their proximal genes and CpG features. Gene annotations and CpG features were obtained from SeqMonk (distributed from Babraham Institute) feature files (which are based on Ensembl annotation). The SeqMonk feature table contains a “biotype”; we used biotype “protein coding”, i.e., we related each iVMF to the nearest protein coding gene. Limits were not imposed on the distance of iVMF from a gene. From the list of iVMFs and analysed fragments, a variability score for features and genes were assigned (number of iVMFs per feature/number of analysed fragments per feature).

### Preparation of RNA from neutrophils and RNA-Seq library construction

For the transcriptomics experiment, neutrophil RNA from four individuals was obtained using previously published protocol (details in Supplementary methods)^79^. The median RNA integrity number (RIN) for the 4 samples was 8.05. RNA libraries were constructed using 1 μg of total RNA with TruSeq stranded mRNA Sample Preparation kit (Illumina) following the manufacturer’s protocol. RNA was sequenced on the Illumina HiSeq 2000 sequencer (Illumina, USA) with a single-ended, 51-bp run producing raw fastq files.

### RNA-Seq data analysis

The RNA-seq reads were mapped to the human genome (assembly GRCh37) using TopHat (v2.0.11)^80^, transcripts were assembled, abundances (Fragments Per Kilo base per Million or FPKM) of transcript were estimated. Logs 2 of the FPKM values were plotted for the 7 neutrophil housekeeping genes^79^ to confirm the expression stability of these genes and minimal variation in gene expression between individuals (Supplementary Fig. S14). Differential expression was examined with Cufflinks (v2.2.0)^81^ package using default parameters^82^. For exon usage analysis, Human gene models were flattened and reads assigned to exon bins and counted using HTSeq (v0.5.4p5)^83^. Differential exon usage was calculated using DEXseq^37^ (v1.8.0) package. DEXseq uses exon counts and compares generalized linear model to a null model with chi-square distribution to test for differential exon usage. Statistical analysis for RNA-Seq data was carried out within the R statistical environment. The graphs for methylation-expression analysis were generated using Lattice (v0.20–27) or hexbin (1.26.3).

### Gene ontology

Gene ontology (GO) term enrichment and functional annotation analyses were done using the Database for Annotation, Visualization and Integrated Discovery (DAVID, v6.7)^84^. Our gene sets were tested against the background of all protein-coding human genes.

### Statistical analysis

To identify the variable fragments (iVMFs), a Chi-square test was used with n-1 degrees of freedom (where n is the number of individuals investigated for a fragment). The Chi-square test treats each CpG result (methylated or unmethylated) as an individual observation. However, the counts for adjacent CpGs in a fragment might be correlated and therefore significance will be overestimated. For this reason we applied a stringent significance level of 0.001 to the Chi-square. To control for the family-wise error rate, the Bonferroni correction was applied and the adjusted cut-off *P-*value for significance was 1.54 × 10^−8^. For Gene–term enrichment analysis *P-*values were calculated with a modified Fisher’s exact test and *P* < 0.05 was considered significant. The enrichment score in the gene-ontology analysis was the geometric mean of all the enrichment *P-*values for each annotation term associated with the gene members in the group. The other statistical tests described in this article (e.g., chromosome wise distribution, calculation of overlap with regulatory feature) were performed using a Chi-square test (based on contingency table) with Yates correction and *P* < 0.05 was considered significant. Association of gene expression and methylation at genomic elements was performed by ANOVA with a Tukey HSD post-hoc test (*P* < 0.05). Hypergeometric test was used to determine overlap with previous studies (*P* < 0.05).

## Additional Information

**Accession codes:** The DNA methylation data generated for this study have been submitted to the NCBI Gene Expression Omnibus under accession number GSE59163. The RNA-Seq data generated for this study have been submitted under accession number GSE59528.

**How to cite this article**: Chatterjee, A. *et al.* Genome-wide DNA methylation map of human neutrophils reveals widespread inter-individual epigenetic variation. *Sci. Rep.* **5**, 17328; doi: 10.1038/srep17328 (2015).

## Supplementary Material

Supplementary Information

Supplementary Information

Supplementary Information

Supplementary Information

Supplementary Information

## Figures and Tables

**Table 1:** Summary of variably methylated genes.

Functional category	Variable genes^1^	Overlap with list of 441 VMGs^2^	Genes^3^
Promoter	29	21	*NSD1, KLHDC7B, PRCP, MED31, LHX8, ENOSF1, DHX32, CMYA5, C2orf84, AL355149.1*
Gene body	240	192	*DHX32, SUSD1, HSPB6, HOXA5, FBXO41, EML3, ZSWIM4, ZNF578, UGGT2, PRRX2*
Enhancer	4	1	*IL4I1, PPP1R26, RP3-377D14.1, ETV3*
Insulator	8	6	*KCNK15, RIN3, ZBTB46, PRRX2, F12, SP6, ANAPC2, MAPK11*

^1^The genes that showed VS of ≥0.5.

^2^The lists of variably methylated genes (VMGs) for a particular region was overlapped with the overall list of 441 genes.

^3^For the promoter and gene body, the top 10 genes with VS of 1.0 are shown. Complete lists are in Supplementary Table S12.
