# Supplementary material for: Genome-wide DNA methylation map of human neutrophils reveals widespread inter-individual epigenetic variation
Source: Sci Rep. 2015 Nov 27;5:17328. doi: 10.1038/srep17328 (PMC4661471; doi:10.1038/srep17328)
Supplement: Supplementary Information [file srep17328-s1.pdf]

Supplementary information for:

Genome-wide DNA methylation map reveals widespread epigenetic variation in healthy individuals

Aniruddha Chatterjee[1, 2], Peter A. Stockwell[3], Euan J. Rodger[1], Elizabeth J. Duncan[2,4], Matthew F Parry[5], Robert J Weeks[1], Ian M. Morison[1, 2]*

[1]Department of Pathology, Dunedin School of Medicine, University of Otago, 270 Great King Street, Dunedin 9054, New Zealand; [2]Gravida: National Centre for Growth and Development, 2-6 Park Ave, Grafton, Auckland 1142, New Zealand; [3]Department of Biochemistry, University of Otago, 710 Cumberland Street, Dunedin 9054, New Zealand; [4]Laboratory for Evolution and Development, Department of Biochemistry, University of Otago, 710 Cumberland Street, Dunedin 9054, New Zealand; [5]Department of Mathematics and Statistics, University of Otago, P.O. Box 56, Dunedin, 9054, New Zealand.

* To whom correspondence should be addressed: Ian Morison, Department of Pathology, Dunedin School of Medicine, University of Otago, P.O. Box 913, Dunedin, New Zealand, Telephone: +64 3 479 7170, E-mail: ian.morison@otago.ac.nz or Aniruddha Chatterjee, Department of Pathology, Dunedin School of Medicine, University of Otago, P.O. Box 913, Dunedin, New Zealand, Telephone: +64 3 470 3455, E-mail: aniruddha.chatterjee@otago.ac.nz

Supplementary Figures S1 - S14,

Supplementary Tables S1 – S9,

Supplementary Boxes S1 - S6

Description of statistical tests to identify inter-individual variably methylated fragments or iVMFs (includes Table S9)

Supplementary methods

Supplementary references

Supplementary Figures

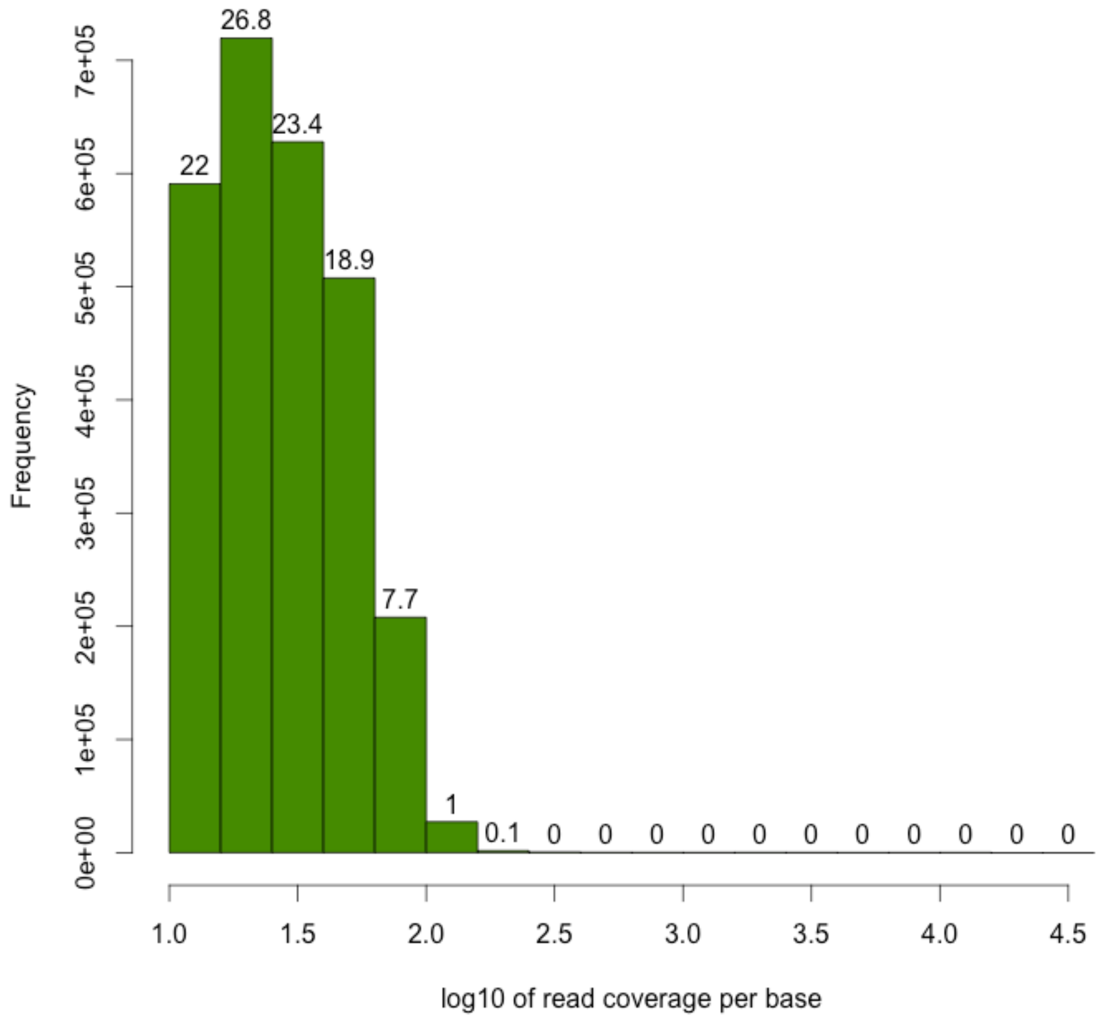

Fig. S1: Representative CpG site coverage histogram of a RRBS library (X9012). The X-axis shows $\log_{10}$ values of the number of reads for each CpG site. The numbers on the bars denote the percentage of CpG site contained in the respective bins. On the X-axis, 1 = 10 reads, 1.5 = 31 reads, 2 = 100 reads, 2.4 = 250 reads, 2.5 = 317 reads, 4 = 10000 reads. For X9012, there were 2.5 million CpG sites that had coverage ≥ 10; the other libraries showed similar histograms.

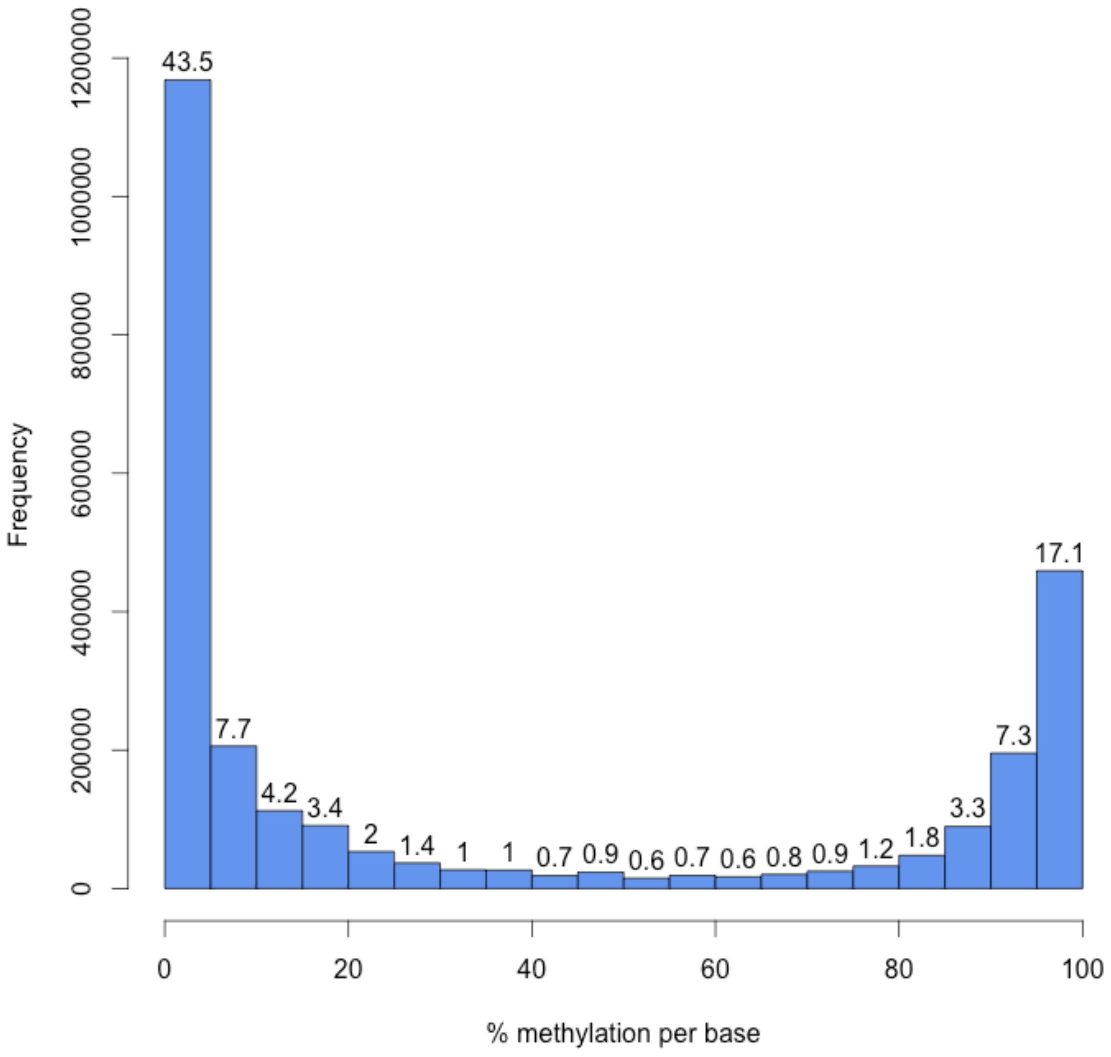

Fig. S2: Representative CpG site methylation histogram of an RRBS library (X9012). The numbers on the bars denote the percentage of CpG sites contained in the respective bins**.** For this sample 1.17 million CpGs (43.5% of the total CpGs) were completely unmethylated (i.e., < 5% methylation) and 0.45 million (17.1% of the total) CpG sites have > 95.0% methylation. The other libraries showed similar distributions.

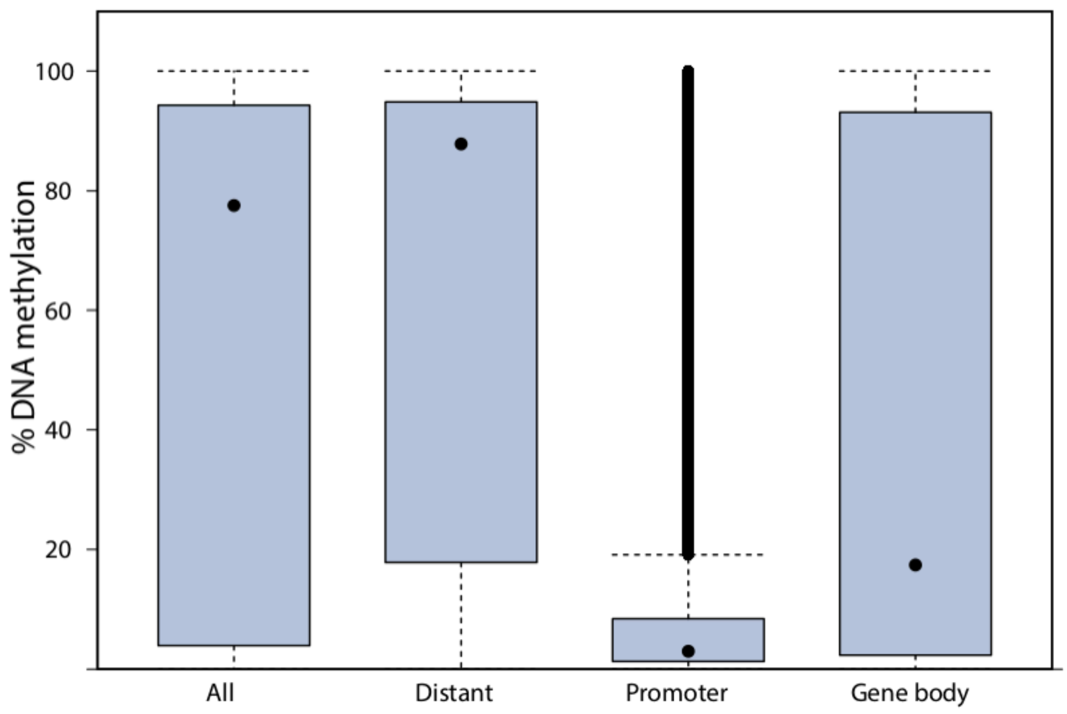

Fig. S3: Tukey boxplot showing the distribution of DNA methylation in different elements of the genome for all analysed samples. Dot = median; top and bottom of box = 1st and 3rd quartile; whiskers = data points within 1.5 times the interquartile range; dots are points outside this range.

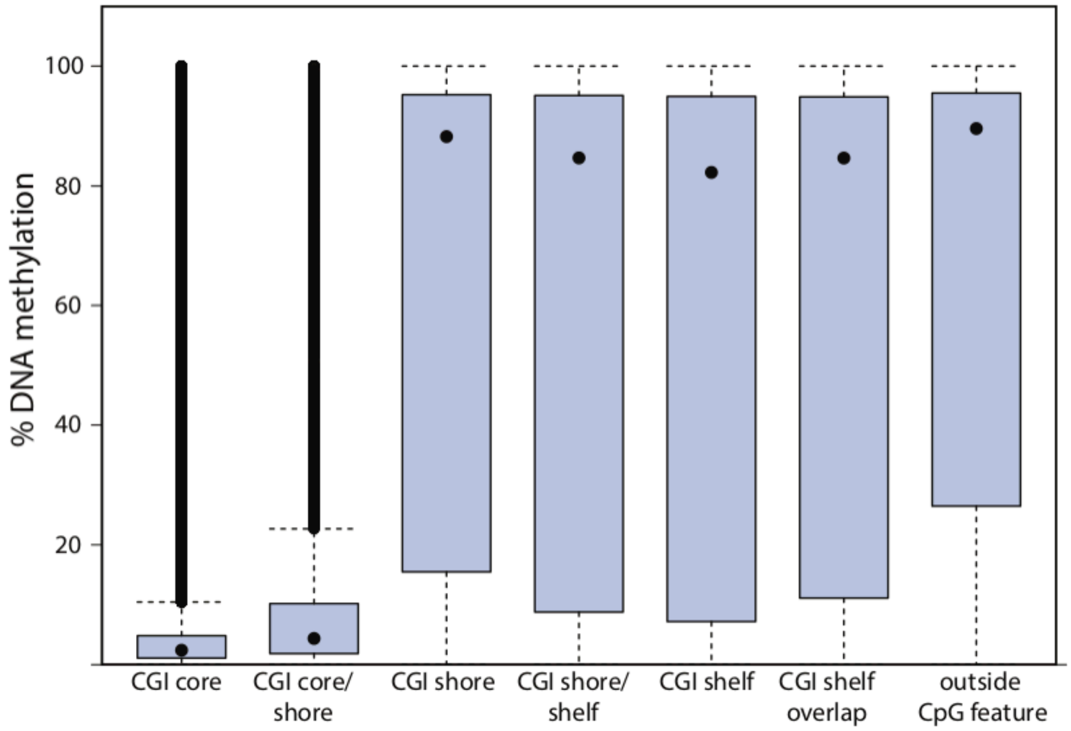

Fig. S4: Boxplot showing the distribution of DNA methylation within each CpG feature and outside CpG features. Dot = median; top and bottom of box = 1st and 3rd quartile; whiskers = data points within 1.5 times the interquartile range; dots are points outside this range.

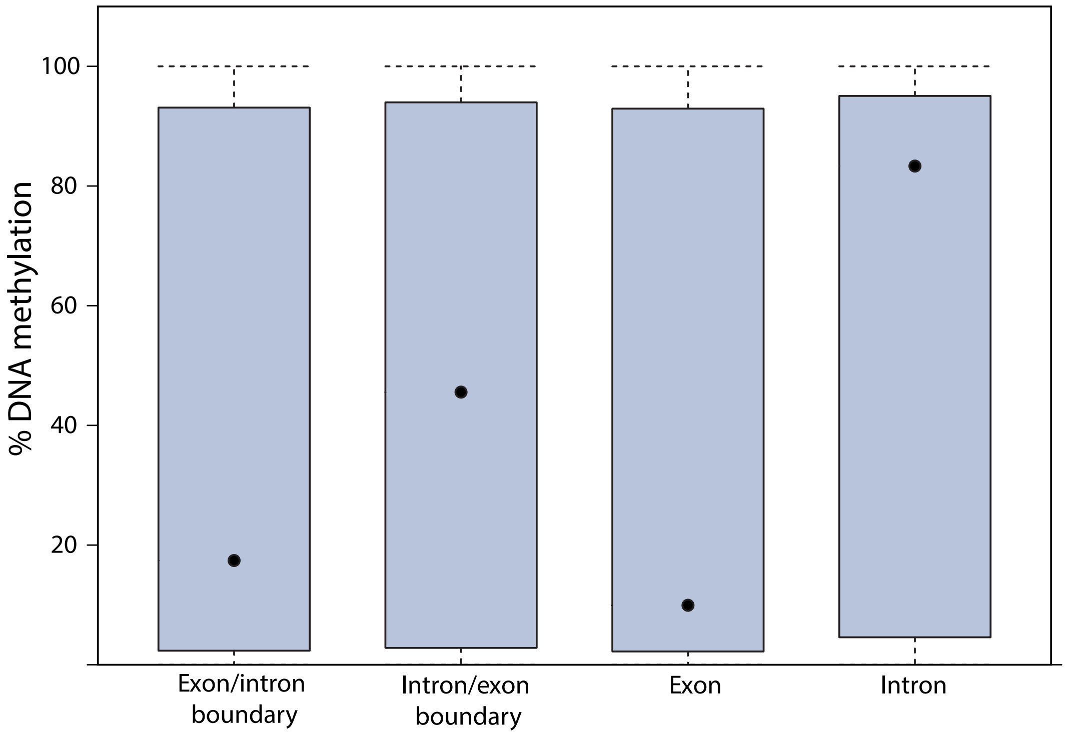

Fig. S5:. Boxplot showing the distribution of DNA methylation in different elements of the gene-body. Dot = median; top and bottom of box = 1st and 3rd quartile; whiskers = data points within 1.5 times the interquartile range; dots are points outside this range.

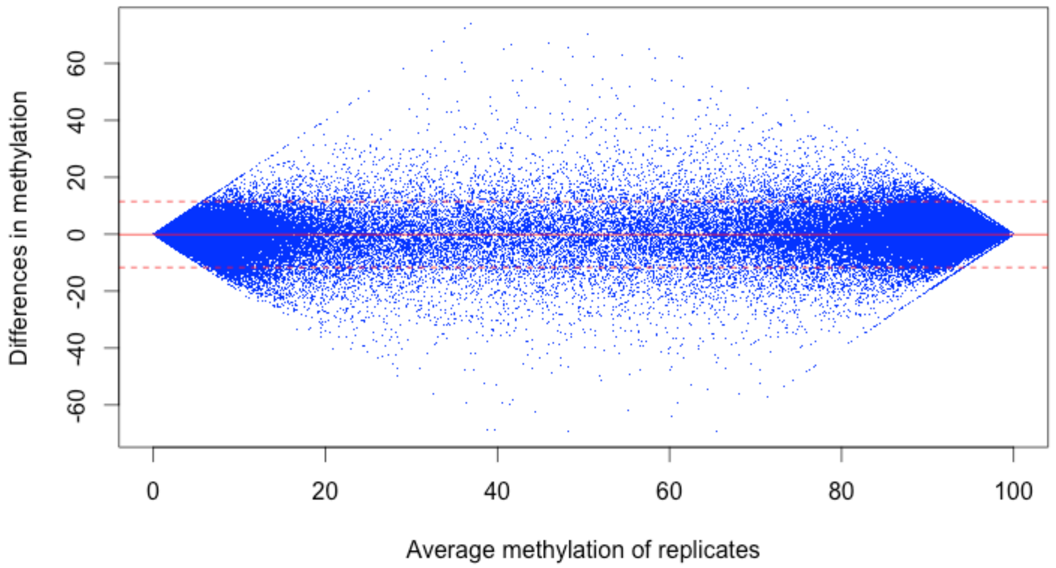

Fig. S6: Bland–Altman plot (mean difference plot) showing the limits of agreement between the technical replicates. The solid red line shows the mean methylation values of the replicates. The top and bottom dotted red lines represents the 95% confidence intervals (mean ± 1.96SD)

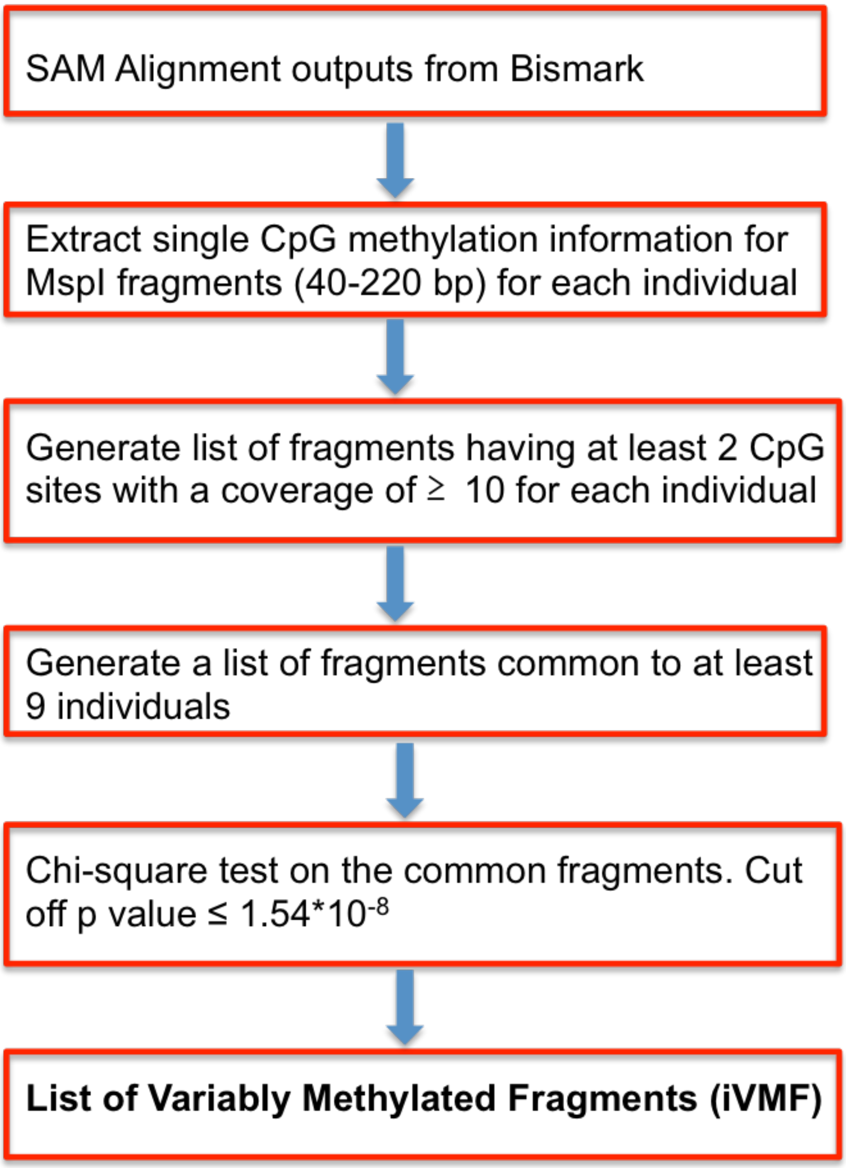

Fig. S7: Pipeline to identify inter-individual variably methylated fragments (iVMFs).

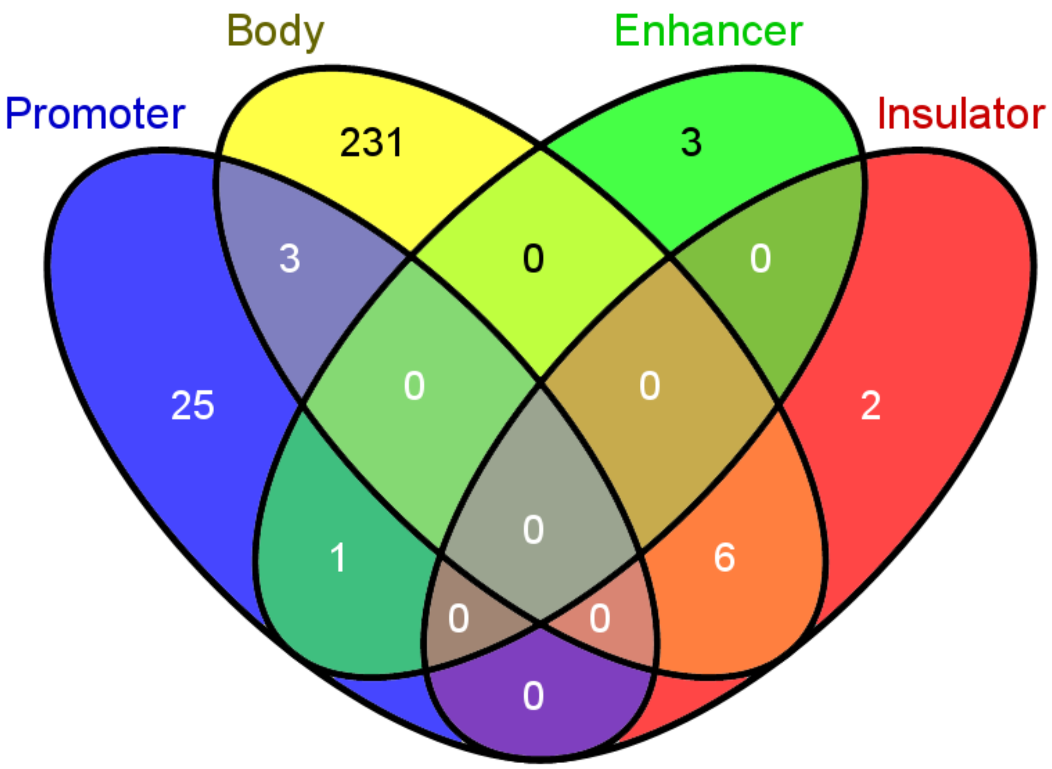

Fig. S8: Venn diagram of overlap of the variably methylated genes that were independently identified in different genomic elements. For each element of the genome (promoter, gene-body, enhancer and insulator) genes that contained ≥ 3 iVMF were analysed and genes with variability score (VS) ≥ 0.5 were considered variable.

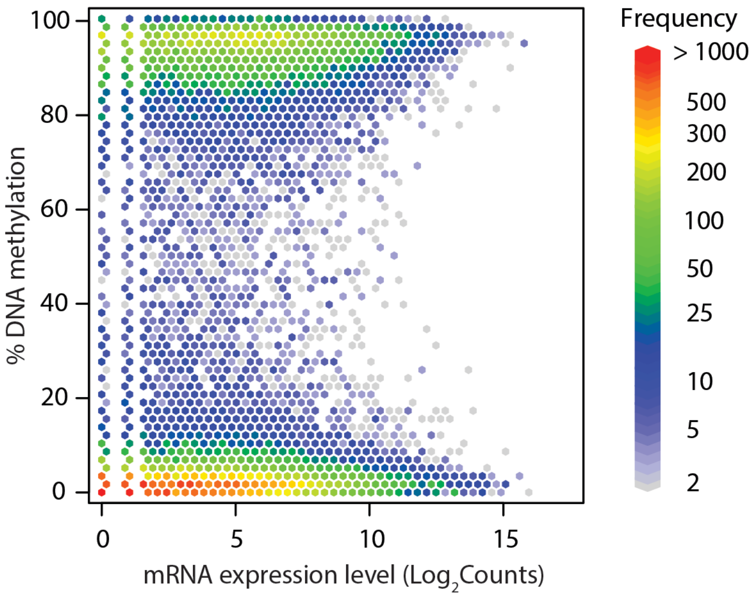

Fig. S9: Hexbin plot illustrating high expression of highly methylated exons. x-axis : counts per exon (log_2 scale). Bins are coloured according to frequency.

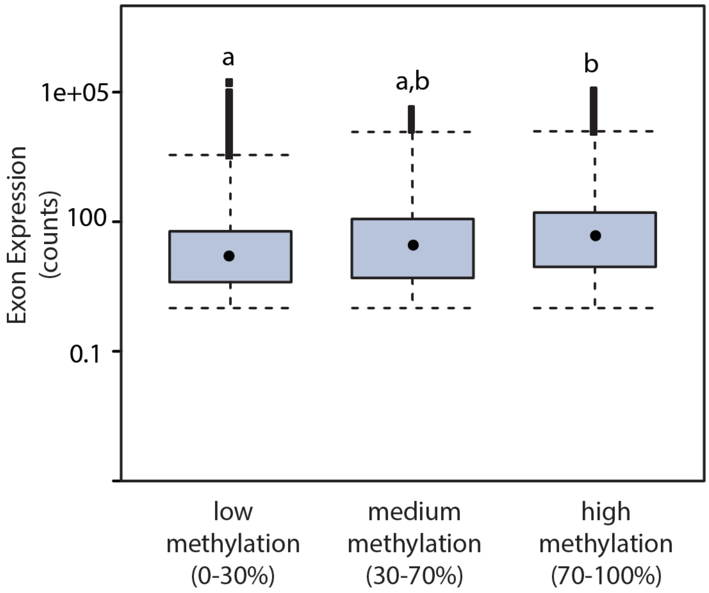

Fig. S10: Box plot demonstrating that high methylation over an exon is associated with higher expression of that exon. Dot = median; top and bottom of box = 1st and 3rd quartile; whiskers = data points within 1.5 times the interquartile range; dots are points outside this range. Bars that do not share a letter are significantly different

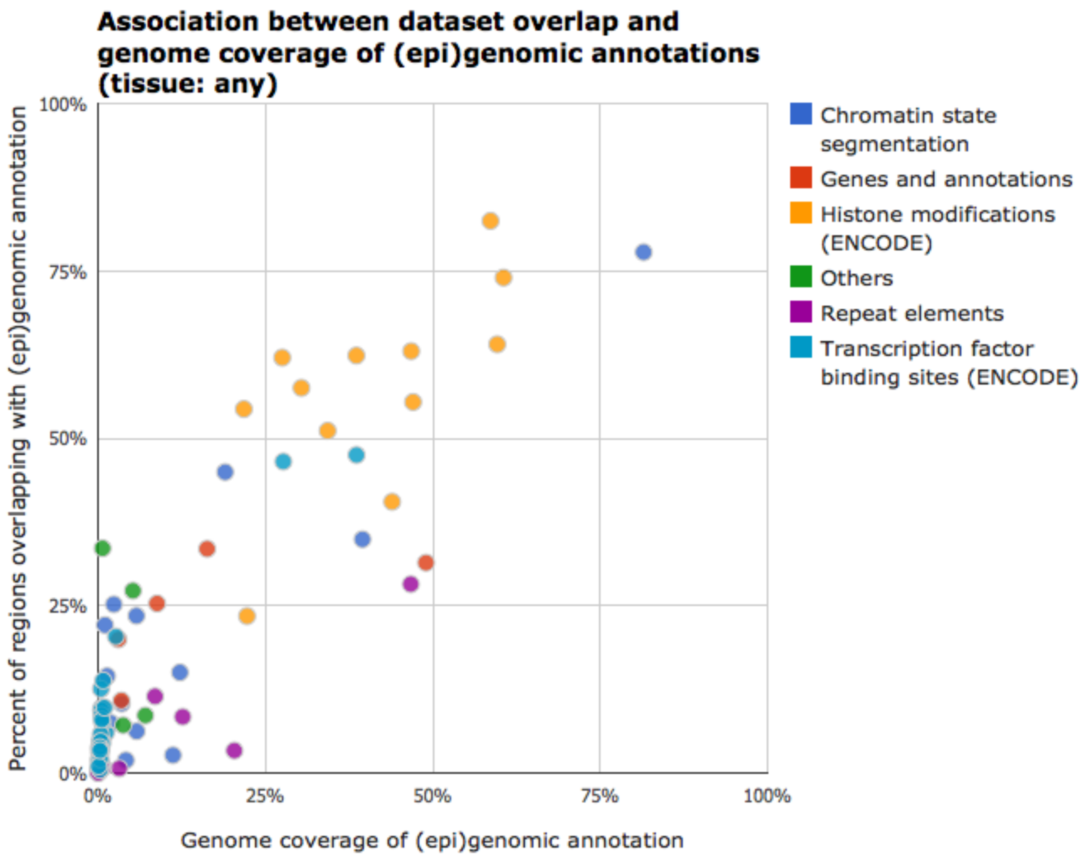

Fig. S11: Summary bubble chart of distant iVMFs of genomic and epigenomic annotations ("any tissue"). This chart is created with EpiExplorer bubble chart feature. Each bubble in the chart represents an element or feature, the X-axis of the chart represents the percentage of overlap of a feature in the genome (genomic coverage) and Y-axis represents the percentage of overlap of that feature in the candidate dataset (epigenomic coverage).

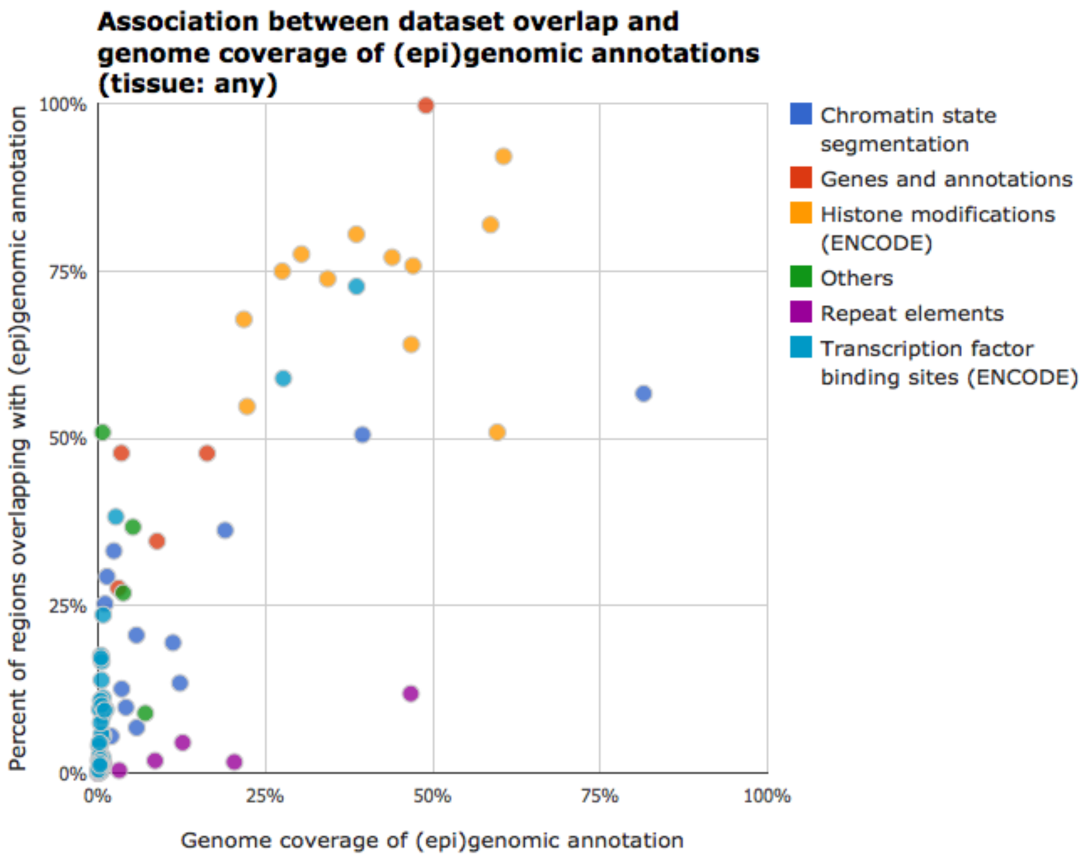

Fig. S12: Summary bubble chart of gene body iVMFs of genomic and epigenomic annotations ("any tissue"). This chart is created with EpiExplorer bubble chart feature. Each bubble in the chart represents an element or feature, the X-axis of the chart represents the percentage of overlap of a feature in the genome (genomic coverage) and Y-axis represents the percentage of overlap of that feature in the candidate dataset (epigenomic coverage).

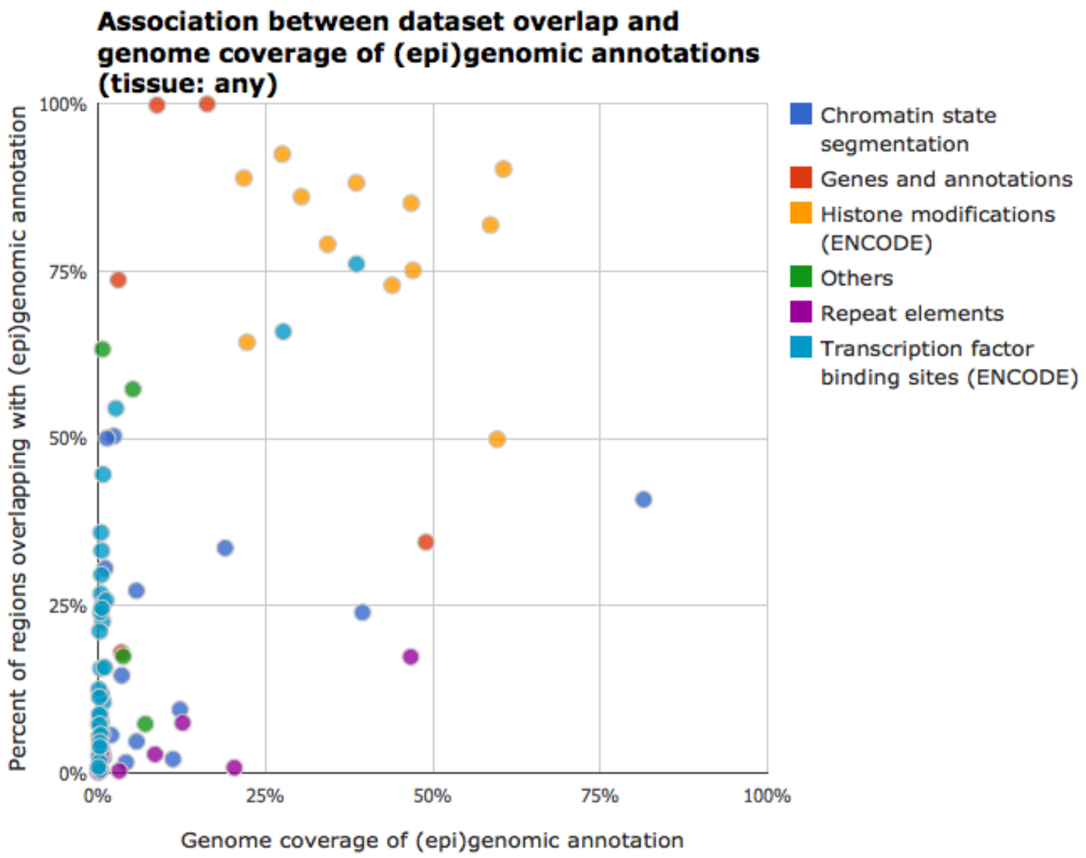

Fig. S13: Summary bubble chart of promoter iVMFs of genomic and epigenomic annotations ("any tissue"). This chart is created with EpiExplorer bubble chart feature. Each bubble in the chart represents an element or feature, the X-axis of the chart represents the percentage of overlap of a feature in the genome (genomic coverage) and Y-axis represents the percentage of overlap of that feature in the candidate dataset (epigenomic coverage).

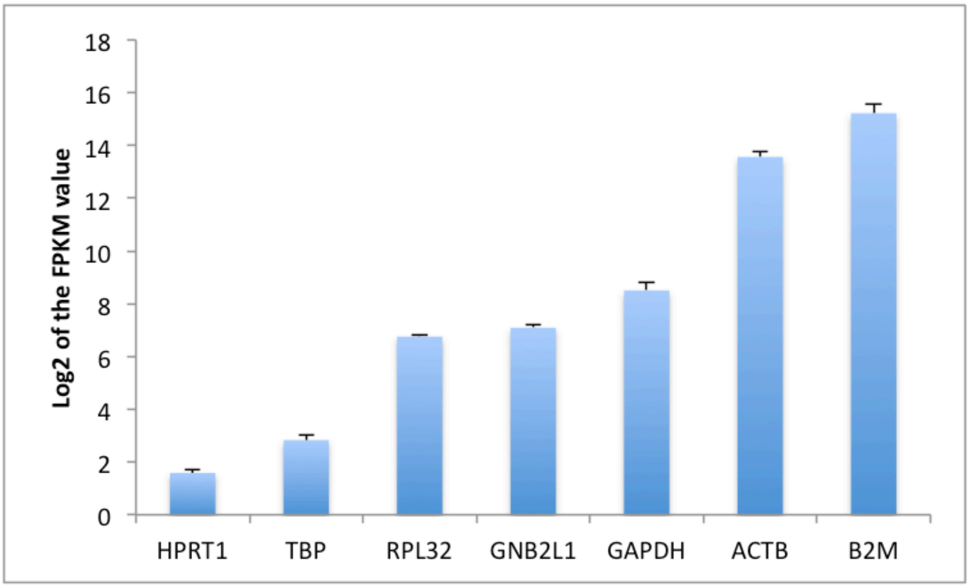

Fig. S14: Stability of gene expression in the analysed individuals for seven housekeeping genes for neutrophils [1]. Error bars show one standard error of the mean (SEM). SEM ranged from 0.07 (RPL32) to 0.33 (B2M) demonstrating stability and minimal variation in expression between individuals.

Supplementary Tables

Table S1. Details of the participants included in the study

Sample ID	Gender	Age	Origin
X9015	Male	26	Indian subcontinent
X9012	Male	29	South America
X9014	Male	31	United Kingdom
X9018	Male	33	Pacific islands
X9020	Male	34	Asia
X9007	Female	25	Indian subcontinent
X9021	Female	28	Pacific islands
X9006	Female	31	Asia
X9016	Female	32	Western Europe
X9019	Female	33	Eastern Europe
X9010	Female	34	United Kingdom

Table S2. Sequenced RRBS library mapping summary#

Sample ID	Sequenced reads (millions)	Length after hard trimming	Processed reads (millions)*	Unique mapping (%)	Multiple mapping (%)	CpG methylation percentage
X9015	18.5	75	18.4	60.7	13.0	39.6
X9006	21.9	65	21.7	55.5	21.7	41.0
X9010	31.1	75	30.9	58.5	21.3	36.2
X9007	9.5	100	9.1	72.4	8.9	34.3
X9019	30.0	80	28.3	67.4	17.0	32.0
X9020	20.8	80	17.9	64.2	14.6	30.0
X9014	21.5	90	18.7	64.7	19.0	28.0
X9012	32.9	85	31.3	68.8	17.8	34.0
X9018	45.7	85	43.8	70.4	14.5	36.6
X9016	49.0	80	45.6	69.0	19.2	35.3
X9021	45.5	80	43.1	66.6	18.6	30.1
X9012 _replicate	17.7	85	16.3	67.5	19.0	37.2

After trimming the 3 filled-in base from the 3' end of the reads. The reads were mapped against the complete human genome GRCh37. The mapping runs were performed on a Mac Pro with 64 bit duo quad core Intel Xeon processors and with 22 Gb RAM running MacOS 10.6 and later MacOS 10.7.
*Reads after QC check and adaptor cleaning.

Table S3. Number of MspI fragments with at least two CpGs with coverage of ≥ 10 in the RRBS cohort

Sample	Total number of fragments	Number of CpG sites[#]
X9012	307712	1996825
X9014	142108	864623
X9018	347536	2138975
X9019	295926	1866592
X9020	176249	1134616
X9021	295312	1826860
X9006	178622	1125614
X9007	115141	827385
X9010	283363	1918256
X9015	271871	1888801
X9016	302622	1848742
X9012 _replicate	187572	1101352

the number of CpG sites contained within those MspI fragments.

Table S4. Overlap of iVMFs and analysed fragments with CpG features

CpG feature[#]	iVMFs (%)	Analysed fragments (%)
CGI core	23.0	24.0
CGI shore	41.0	39.9
CGI shelf	1.3	1.32
Core_shore overlap	0.90	0.91
Shore_shelf overlap	0.03	0.04

[#] CGI= CpG island.

Table S5. Mean of CpGs per fragment by genomic regions: iVMFs vs. Non-variable fragments.

Features	iVMFs (CpG/Fragment)	Non variable (CpG/Fragment)	P-value
Promoter	8.6	7.46	0.0001
Gene body	7.32	6.68	0.0001
Distant	6.61	5.92	0.0001

Table S6. Frequency distribution of genes associated with iVMFs

**Number of associated iVMFs**	**Number of genes**	**Number of novel genes**[#]
30-35	2	1
25-30	3	2
20-25	8	7
15-20	17	12
10-15	53	44
5-10	397	330
4	299	257
3	519	466
2	1342	1249
1	3712	3500

[#] These genes did not appear in Feinberg, Harris or Waterland's lists of metastable genes.

Table S7. Relationship of gene expression and methylation of promoters

Promoter definition	Low methylation (median FPKM)	Medium Methylation (median FPKM)	High methylation (median FPKM)	*P*-value
0 to + 500	1.403	0.075	0.00	3.5e-16
-200 to + 500	1.303	0.068	0.00	3.13e-14
-500 to + 500	1.253	0.125	0.00	4.36e-12
-1000 to + 500	1.182	0.185	0.025	8.56e-08
-1500 to + 500	1.128	1.128	0.065	9.79e-07
-2000 to + 500	1.093	0.184	0.107	1.04e-05
-3000 to + 500	1.043	0.160	0.162	0.000276
-5000 to + 500	0.971	0.148	0.223	0.402

Table S8. Gene ontology term enrichment of 281 iVMF associated genes[1]

Annotation term	Number of genes	P value[2]
**Biological Process**		
Regulation of exocytosis	4	0.0099
Locomotory behavior	10	0.0104
Regulation of transcription	47	0.0179
Metal ion transport	13	0.0201
Embryonic organ morphogenesis	6	0.0312
Gland development	6	0.0329
Chromatin remodeling	4	0.0373
**Molecular Function**		
Ion channel activity	15	0.0009
Zinc ion binding	43	0.0321
Transcription regulator activity	30	0.0408
**Cellular Compartment**		
Contractile fiber	6	0.0207
Cell junction	13	0.0376
Synapse	10	0.0421
**Protein Domain**		
Groucho/transducin-like enhancer	3	0.0018
Ion transport	7	0.0042
Leucine-rich repeat	8	0.0287
Winged helix repressor DNA-binding	7	0.0455
**Tissue**		
Brain	128	0.0006
Cartilage	3	0.0450

[1] DAVID was used for gene ontology enrichment of 256 unique gene IDs associated with 281 iVMFs (variation score > 0.5).
[2] DAVID uses a modified Fisher's exact test for enrichment evaluation analysis.

Supplementary Boxes

Supplementary Box S1. Overlap of genes between this study and Waterland et al. [2]

Common variably methylated genes between Waterland *et al* and our findings
*PAX8**, *ZFYVE28*

* *PAX8* had variable score of 0.41 so this is not one of the 441 top variably methylated genes.

Supplementary Box S2. Overlap of genes between this study and Harris et al [3].

48 common variably methylated genes between Harris *et al* (1013 genes) and our findings (441 genes)
FRG2C, KCNK15, ZNF714, SLC2A9, FCGR3A, ADAMTS14, ZNF385A, WDR27, VWF, CCDC102A, MEST, C2orf27B, VPS53, RGPD8, CYP2E1, C21orf56, C10orf25, AGTR1, C8orf73, PARD6G, ZFYVE28, ZC3H12D, TFAP2E, KCNA3, CCDC144NL, C6orf145, C19orf77, C7orf50, RPH3AL, NPAS3, OLIG1, CCBE1, SYTL1, RFPL2, IFITM5, C18orf1, PANX2, SH3BP2, POMC, PPP2R2A, GALNT8, C10orf47, UNC5B, TRIM27, TNIP2, PARVB, NOP14, ATHL1

Supplementary Box S3. Overlap of genes between this study and Feinberg et al [4].

6 common variably methylated genes between Feinberg *et al* (212 genes) and our findings (441 genes)
HOXA5, TACSTD2, INSIG2, NXN, MEST, RPH3AL

Supplementary Box S4. Overlap of genes between this study, Harris and Feinberg et al

Common variably methylated genes between Harris *et al* and Feinberg et al (15 genes)
ARPP-21, BCL11B, BCOR, GPR123, GPR85, GRIN1, KIAA1009, MAD1L1, MEST, MOG, NDUFA3, PTPRN2, RPH3AL, TNFSF9, TRIM31

Supplementary Box S5. Overlap of genes between this study, Waterland and Harris et al

Common variably methylated genes between Waterland *et al*, Harris et al and our findings (1 gene)
ZFYVE28

Supplementary Box S6. Overlap of genes between this study, Harris and Feinberg et al

Common variably methylated genes between Harris et al, Feinberg et al and our findings (2 genes)
MEST, *RPH3AL*

Description of statistical test to identify inter-individual variably methylated fragments (iVMFs)

Two statistical tests were widely used to identify variable (or differential) methylation patterns. They are Fisher's Exact and Chi-square statistics. Fisher's Exact is a widely used statistical method in epigenetics study where differential methylation is assessed over a sliding or fixed window on a pairwise basis and by applying fold-differences in methylation between the groups[5]. For the current analysis, in which 11 individuals were involved, application of Fisher's Exact meant the output would contain 55 different pairwise tests and the P-values for all possible pairs among 11 individuals. For a given MspI fragment, pairwise Fisher's Exact tests can be performed between multiple samples and the lowest probability taken to indicate the extent differential methylation, although this may obscure a number of insignificant differences between other samples. For two group or pairwise comparison, e.g., disease vs. control, Fisher's Exact is a good approach. To investigate methylation across multiple samples, however, the preferred statistics was a single Chi-square statistic for all samples that meet Chi-square requirements of expected counts ≥ 5.0. The test computed the observed and expected counts of methylated (+) and unmethylated (-) CpG

sites for each fragment in a 2 x 11 contingency table and produced a single P-value for each fragment. Various thresholds can be applied to restrict the tests to fragments and samples that meet criteria for CpG number, adequate density of CpG mapping and statistical significance (threshold P-values).

In a Chi-square test it is possible that different patterns of methylation variation might be scored as significant. In an ideal scenario, 11 individuals will show different methylation counts from each other and therefore a fragment will qualify as iVMF. However, it is possible that in some cases one or two individual will contribute substantially to the overall probability of the fragment, i.e., out of 11 individuals, nine of them might show similar methylation counts, whereas two individuals might have dissimilar methylation. We did not want to exclude this possibility as this might reflect rare vs common phenotypic variation.

After performing the Chi-square test, a Bonferroni correction was applied for multiple comparison to derive a cut-off p value for filtering iVMFs (at a significance level of 0.001, the adjusted p value cut off after Bonferroni correction was 1.54 $x10^{-8}$). A general criticism of Bonferroni correction is that the test is extremely stringent and only controls for Type I errors (false positives), therefore increasing the number of false negatives. Several other methods (such as False Discovery rate and Holms method) have been applied in biological statistics to address this issue. Table S9 shows a comparative example of commonly used multiple test correction methods (note this table was based on simulated values not on real data to show the nature of different multiple correction tests). The comparison reveals that the number of significant observations was least when Bonferroni was applied compared to Holms and FDR method.

Table S9. Comparisons of common multiple correction tests

Test number (i)	p-value (ordered)	Bonferroni (α/n)	Holm (α/(n-i+1)	FDR (iα/n)	Unadjusted α= 0.05
1	0.002	0.00625*	0.00625*	0.00625*	0.05*
2	0.004	0.00625*	0.00714*	0.01250*	0.05*
3	0.007	0.00625	0.00833*	0.01875*	0.05*
4	0.01	0.00625	0.01000*	0.02500*	0.05*
5	0.02	0.00625	0.01250	0.03125*	0.05*
6	0.03	0.00625	0.01667	0.03750*	0.05*
7	0.05	0.00625	0.02500	0.04375	0.05*
8	0.08	0.00625	0.05	0.05000	0.05
Number of significant		2	4	6	7
Expected Type I errors#		0.05	0.05	<0.3(0.075)	0.4

*Statistically significant. # Type I error means false positive, i.e., when the observation was not significant and the null hypothesis is true but erroneously null hypothesis is rejected. (Courtesy: Dr. Micheal Black)

While performing a very large number of tests, the stringency of the Bonferroni correction could result in to an extremely low adjusted p value and consequently the number of significant observations derived from the experiments will be very low. So, applying the Bonferroni correction could lead to the loss of many fragments which would have been qualified as a variable one if other correction tests (such as FDR) were applied. However, when we performed analysis on the Chi-square values of the fragments, we found even after applying stringent Bonferroni correction, a large number of fragments were significant. Therefore, we reasoned that we would apply this stringency to minimise false positives. We might have got more fragments by using less stringent criteria or less stringent test, but we decided to select only the highly significant ones. A post-hoc analysis of the variable fragments obtained by more relaxed correction criteria will remain a subject of future research.

Chi-square test was chosen in advance as the distribution of methylation (e.g., normal, bimodal or trimodal) was not known. Our data provides

opportunities for additional analyses and simulation of the data to other researchers that might help to inform choices of statistical tests for DNA methylation analysis.

Supplementary Methods:

Neutrophil isolation: 16 mL EDTA-anticoagulated blood was diluted (1:1) in phosphate buffered saline (PBS) layered on Ficoll-Paque PLUS (GE Healthcare) and centrifuged at 400 × g for 40 min at RT. The plasma layer and the "mononuclear layer" were discarded. A neutrophil-rich pellet was obtaining by twice lysing red blood cells in 0.17 M NH_4Cl for 30 min followed by centrifugation at 300 × g for 10 min. The pellet was resuspended in 2 mL PBS. The processed samples contained no dead cells, as assessed by trypan blue staining. Cells were counted (Sysmex XE2100) after neutrophil enrichment and only neutrophil enriched samples (> 90%) were used for subsequent DNA extraction.

RRBS library preparation: Briefly, genomic DNA was digested overnight with MspI, followed by end-repair, addition of 3'-A overhangs and ligation of methylated adaptors (Illumina, San Diego, CA). 40 to 220 bp fragments (pre-adaptor-ligation size) were excised from 3% Nusieve agarose gels (Lonza, Basel, Switzerland) and bisulfite-converted (EZ DNA methylation kit, Zymo Research, Irvine, CA). Bisulfite-converted libraries were amplified by PCR (16-18 cycles). The final RRBS library was assessed using the Qubit fluorometer (Life Technologies, Grand Island, NY) and the 2100 Bioanalyzer (Agilent Technologies, Palo Alto, CA). The technical replicate library was prepared with the same DNA material (sample: X9012) and TruSeq (Illumina) kit but was sequenced in a different flow cell.

Quality assessment, processing and alignment of sequenced reads: Quality of the sequenced reads for each individual sample was assessed

using the FastQC (http://www.bioinformatics.babraham.ac.uk/projects/fastqc/) program. Trimming of the sequenced reads was performed where median Phred score of the sequences were < 30 (=0.001 probability of a base call error in the Illumina base-calling pipeline) with fastq_quality_trimmer v0.0.13 tool (http://hannonlab.cshl.edu/fastx_toolkit/). Adaptor sequences were removed from the reads using our in-house cleanadaptors program[6]. Results from the CpG bases at the 3' end of the reads were removed since these do not reflect the native state of the input DNA. The sequenced reads were aligned against the complete human reference genome GRCh37 with Bismark v0.6.4 alignment tool[7] with a stringent criteria of one mismatch (default=2) in the seed (i.e., in the first 28 bp of the sequenced reads). The alignments were performed on a Mac Pro with 64 bit duo quad core Intel Xeon processors and with 22 Gb RAM running MacOS 10.6.

Analysis of technical replicates and visualization of RRBS data: For assessment of the technical replicates, CpG sites with 10 or more sequenced reads in each library were included. Differential methylation analysis of these sites (1.3 million CpG sites) was performed using R package, methylKit[8]. For visualization of methylation, the aligned SAM files were numerically sorted by chromosome number and position and imported in the integrated genome viewer (IGV)[9]. The reads were visualised in bisulfite mode with only the CG track on.

Preparation of RNA from neutrophils: For each participant 20 mL of peripheral blood was collected into heparinized tubes. Enrichment for neutrophil was performed by Dextran-Ficoll sedimentation and centrifugation[1]. Each preparation contained > 98% neutrophils. Total RNA was isolated using the RNeasy Mini Kit (Qiagen) following manufacturer's protocol. Two rounds of RNase-free DNase I digestion (Qiagen) was performed to remove genomic DNA during the extraction process. RNA concentrations were determined using NanoDrop 2000 (Thermo Scientific, MA, USA). The quality of the RNA was determined using the RNA 6000 Pico chip on 2100 Bioanalyzer (Agilent

Technologies). The median RNA integrity number (RIN) for the 4 samples was 8.05. RNA libraries were constructed using 1 μg of total RNA with TruSeq stranded mRNA Sample Preparation kit (Illumina) following the manufacturer's protocol. RNA was sequenced on the Illumina HiSeq 2000 sequencer (Illumina, USA) with a single-ended, 51-bp run producing raw fastq files.

Supplementary references:

1 Zhang, X., Ding, L. & Sandford, A. J. Selection of reference genes for gene expression studies in human neutrophils by real-time PCR. *BMC Mol Biol* **6**, 4, doi:10.1186/1471-2199-6-4 (2005).

2 Waterland, R. A. *et al.* Season of conception in rural gambia affects DNA methylation at putative human metastable epialleles. *Plos Genetics* **6**, e1001252, doi:10.1371/journal.pgen.1001252 (2010).

3 Harris, R. A., Nagy-Szakal, D. & Kellermayer, R. Human metastable epiallele candidates link to common disorders. *Epigenetics : Official Journal Of The Dna Methylation Society* **8** (2013).

4 Feinberg, A. P. *et al.* Personalized epigenomic signatures that are stable over time and covary with body mass index. *Sci Transl Med* **2**, 49ra67, doi:2/49/49ra67 [pii]
10.1126/scitranslmed.3001262 (2010).

5 Li, Y. *et al.* The DNA methylome of human peripheral blood mononuclear cells. *Plos Biol* **8**, e1000533, doi:10.1371/journal.pbio.1000533 (2010).

6 Chatterjee, A., Stockwell, P. A., Rodger, E. J. & Morison, I. M. Comparison of alignment software for genome-wide bisulphite sequence data. *Nucl. Acids Res.* **40**, e79, doi:10.1093/nar/gks150 (2012).

7 Krueger, F. & Andrews, S. R. Bismark: a flexible aligner and methylation caller for Bisulfite-Seq applications. *Bioinformatics* **27**, 1571-1572, doi:10.1093/bioinformatics/btr167 (2011).

8 Akalin, A. *et al.* methylKit: a comprehensive R package for the analysis of genome-wide DNA methylation profiles. *Genome Biology* **13**, R87, doi:10.1186/gb-2012-13-10-r87 (2012).

9 Thorvaldsdottir, H., Robinson, J. T. & Mesirov, J. P. Integrative Genomics Viewer (IGV): high-performance genomics data visualization and exploration. *Briefings In Bioinformatics*, doi:10.1093/bib/bbs017 (2012).
